# Loss of Extreme Long-Range Enhancers in Human Neural Crest Drives a Craniofacial Disorder

**DOI:** 10.1016/j.stem.2020.09.001

**Published:** 2020-11-05

**Authors:** Hannah K. Long, Marco Osterwalder, Ian C. Welsh, Karissa Hansen, James O.J. Davies, Yiran E. Liu, Mervenaz Koska, Alexander T. Adams, Robert Aho, Neha Arora, Kazuya Ikeda, Ruth M. Williams, Tatjana Sauka-Spengler, Matthew H. Porteus, Tim Mohun, Diane E. Dickel, Tomek Swigut, Jim R. Hughes, Douglas R. Higgs, Axel Visel, Licia Selleri, Joanna Wysocka

**Affiliations:** 1 Department of Chemical and Systems Biology, Stanford University School of Medicine, Stanford, CA 94305, USA; 2 Department of Developmental Biology, Stanford University School of Medicine, Stanford, CA 94305, USA; 3 Institute of Stem Cell Biology and Regenerative Medicine, Stanford University School of Medicine, Stanford, CA 94305, USA; 4 Environmental Genomics and Systems Biology Division, Lawrence Berkeley National Laboratory, Berkeley, CA 94720, USA; 5 Program in Craniofacial Biology, Department of Orofacial Sciences and Department of Anatomy, Institute of Human Genetics, Eli and Edythe Broad Center of Regeneration Medicine and Stem Cell Research, University of California, San Francisco, San Francisco, CA, USA; 6 MRC Molecular Haematology Unit, MRC Weatherall Institute of Molecular Medicine, Radcliffe Department of Medicine, University of Oxford, Oxford, UK; 7 Cancer Biology Program, Stanford University School of Medicine, Stanford, CA 94305, USA; 8 Department of Biology, Stanford University, Stanford, CA 94305, USA; 9 Department of Pediatrics, Stanford University, Stanford, CA 94305, USA; 10 MRC Weatherall Institute of Molecular Medicine, Radcliffe Department of Medicine, University of Oxford, Oxford, UK; 11 The Francis Crick Institute, Mill Hill Laboratory, The Ridgeway, Mill Hill, London NW7 1AA, UK; 12 Laboratory of Gene Regulation, MRC Weatherall Institute of Molecular Medicine, Radcliffe Department of Medicine, University of Oxford, Oxford, UK; 13 US Department of Energy Joint Genome Institute, Lawrence Berkeley National Laboratory, Berkeley, CA 94720, USA; 14 School of Natural Sciences, University of California, Merced, Merced, CA 95343, USA; 15 Howard Hughes Medical Institute, Stanford University School of Medicine, Stanford, CA 94305, USA

**Keywords:** enhancer, enhanceropathy, Pierre Robin sequence, *SOX9*, gene dosage, long-range regulation, neural crest, craniofacial, transcription, non-coding mutation

## Abstract

Non-coding mutations at the far end of a large gene desert surrounding the *SOX9* gene result in a human craniofacial disorder called Pierre Robin sequence (PRS). Leveraging a human stem cell differentiation model, we identify two clusters of enhancers within the PRS-associated region that regulate *SOX9* expression during a restricted window of facial progenitor development at distances up to 1.45 Mb. Enhancers within the 1.45 Mb cluster exhibit highly synergistic activity that is dependent on the Coordinator motif. Using mouse models, we demonstrate that PRS phenotypic specificity arises from the convergence of two mechanisms: confinement of *Sox9* dosage perturbation to developing facial structures through context-specific enhancer activity and heightened sensitivity of the lower jaw to *Sox9* expression reduction. Overall, we characterize the longest-range human enhancers involved in congenital malformations, directly demonstrate that PRS is an enhanceropathy, and illustrate how small changes in gene expression can lead to morphological variation.

## Highlights

• Extreme long-range enhancer clusters overlap PRS patient mutations at the *SOX9* locus
• PRS enhancers drive stage-specific *SOX9* expression in the cranial neural crest
• Mandible development has heightened sensitivity to perturbation of *SOX9* gene dosage
• Deletion of mouse EC1.45 leads to quantitative changes in mandible morphology

## Graphical Abstract

**Figure:**
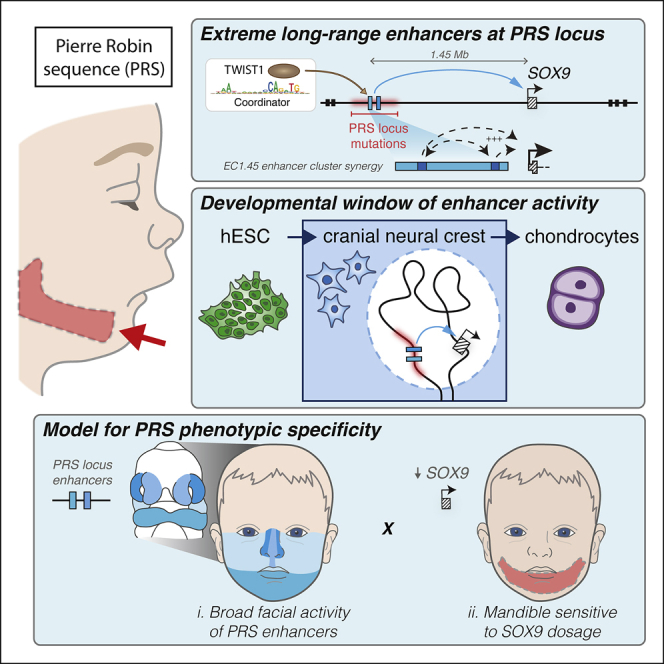

## Teaser

Non-coding mutations over a megabase from *SOX9* cause the craniofacial disorder Pierre Robin sequence (PRS). Long et al. leverage a human neural crest model to demonstrate that PRS is caused by loss of extreme long-range enhancers active during a restricted developmental window and explore mechanisms underlying the specificity of disease manifestations.

## Introduction

Distal regulatory sequences called enhancers control gene transcription at a distance and play a critical role in directing developmental gene expression patterns (Long et al., 2016). Non-coding mutations are increasingly being implicated in human disease (Franke et al., 2016; Laugsch et al., 2019; Lupiáñez et al., 2015), and, in particular, perturbations of enhancers have been documented as being causative because of their effects on gene regulation during development (Spitz, 2016). Although mutations of protein-coding sequences often affect multiple tissues in which a given gene is active, mutations in non-coding regulatory regions can selectively perturb target gene expression in specific tissue contexts. For example, SOX9, an SRY (sex-determining region Y)-related HMG (high mobility group) box (SOX) transcription factor, plays numerous important roles during embryogenesis, including sex determination, chondrogenesis, and craniofacial development (Lee and Saint-Jeannet, 2011; Lefebvre and Dvir-Ginzberg, 2016). Heterozygous loss-of-function mutations in the *SOX9* coding sequence cause a severe congenital disorder called campomelic dysplasia, which is associated with bowed long limbs, disorders of sex determination, and craniofacial defects (Wagner et al., 1994). Interestingly, *SOX9* is the sole protein-coding gene within an unusually large, ~2-Mb topologically associating domain (TAD) (Bagheri-Fam et al., 2006; Gordon et al., 2009). Many non-coding mutations have been described within this gene desert, including large deletions, translocations, and duplications, that cause a range of defects that recapitulate distinct aspects, but not all features, of campomelic dysplasia, leading to the hypothesis that cell-type-specific enhancers are disrupted in these tissue-selective disorders (Baetens et al., 2017; Kurth et al., 2009; Sanchez-Castro et al., 2013). In some cases, the perturbed enhancers have been mapped and characterized; for example, an SRY-responsive regulatory element essential for sex determination (Gonen et al., 2018).

A cluster of large genomic deletions and translocation breakpoints at the centromeric far end of the *SOX9* TAD are associated with isolated Pierre Robin sequence (PRS), a congenital craniofacial disorder characterized by a single primary phenotype: underdevelopment of the lower jaw or mandible (micrognathia) that leads to secondary phenotypes, including retraction of the tongue (glossoptosis), obstruction of the airway, and, with incomplete penetrance, horseshoe-shaped cleft palate (Robin, 1994; Tan and Farlie, 2013). This sequence of anomalies, in turn, results in feeding and breathing difficulties and failure to thrive (Rathé et al., 2015). It has been proposed that PRS-associated mutations perturb the function of key *SOX9* long-range enhancers active during craniofacial development (Amarillo et al., 2013; Benko et al., 2009; Gordon et al., 2009, 2014); however, functional characterization of putative craniofacial enhancers and direct demonstration that *SOX9* is the target gene are still lacking. Given the specificity of the developmental defects in PRS and the well-documented requirement for SOX9 function in the neural crest (Cheung and Briscoe, 2003; Mori-Akiyama et al., 2003; Spokony et al., 2002), we hypothesized that the centromeric far end of the *SOX9* TAD harbors enhancers active in cranial neural crest cells (CNCCs), a transient population of multipotent progenitor cells that give rise to the majority of vertebrate craniofacial structures, including the jaw (Bronner and LeDouarin, 2012; Minoux and Rijli, 2010; Trainor et al., 2003).

Leveraging a well-characterized *in vitro* differentiation model of human CNCCs (hCNCCs) (Bajpai et al., 2010; Prescott et al., 2015; Rada-Iglesias et al., 2012), we uncover two clusters of hCNCC-specific enhancers overlapping PRS mutations and demonstrate that they regulate *SOX9* transcription within a defined developmental window and over extremely large genomic distances of 1.45 Mb and 1.25 Mb, respectively. To model the sensitivity of craniofacial development to changes in *Sox9* gene dosage, we generate an allelic series in mice with increasing severity of *Sox9* perturbation. We propose a mechanism of disease etiology where two features of *Sox9* regulation converge to confine disease phenotypes to the lower jaw. First, loss of the tissue-specific activity of PRS locus enhancers restricts *Sox9* dosage perturbation to the developing facial structures, and second, heightened sensitivity of the lower jaw to *Sox9* level reduction further confines PRS-associated malformations.

## Results

### Three Clusters of Candidate Human Cranial Neural Crest Enhancers Overlap Sequences Lost in PRS

**Figure 1:**
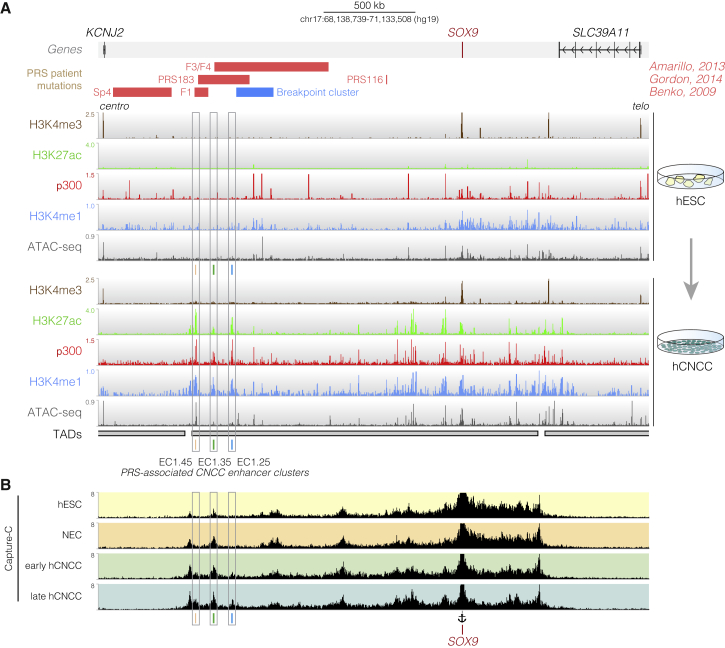
Human Cranial Neural Crest-Specific Enhancers Are Associated with PRS Patient Mutations (A) ChIP-seq and ATAC-seq for hESCs (top) and P4 hCNCCs (bottom) at the human *SOX9* locus. Three putative hCNCC-specific ECs overlap the PRS locus: EC1.45, EC1.35, and EC1.25. PRS patient deletions (red), translocation breakpoints (blue), topological domains (TADs) (Dixon et al., 2012), and protein-coding genes are shown. Centro, centromeric; telo, telomeric. (B) Capture-C from the *SOX9* promoter (see anchor) in hESCs, neuroectodermal spheres (NECs), early (day 11) and late (P4) hCNCCs. See also Figures S1 and S2.

**Figure 2:**
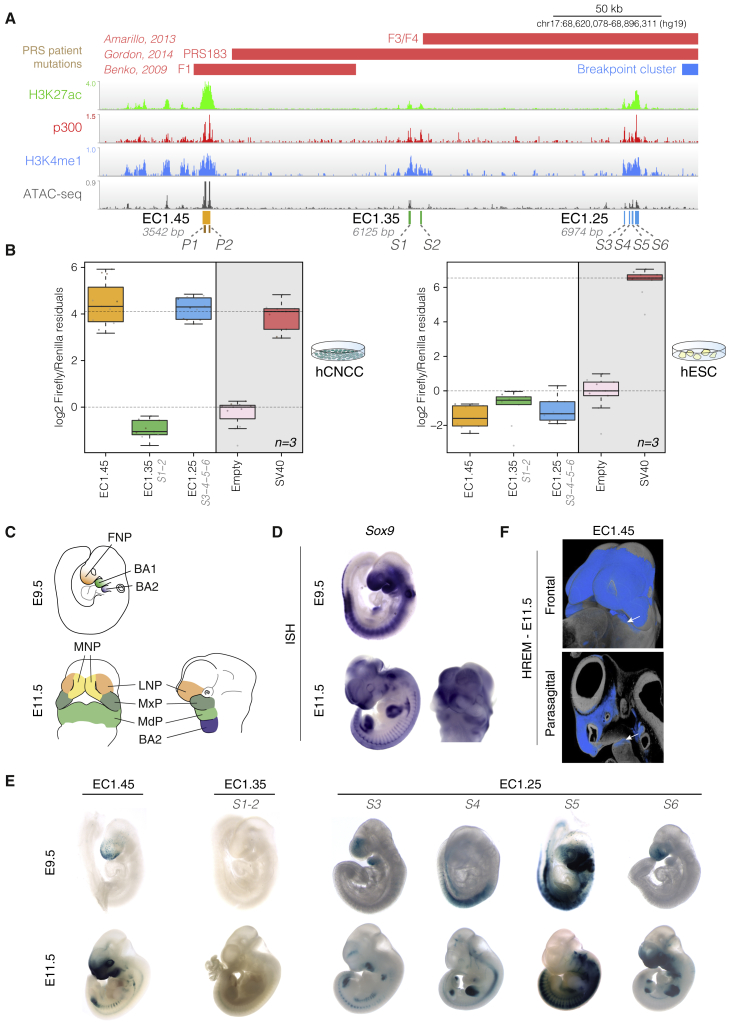
PRS Locus EC1.45 and EC1.25 Are Active in hCNCCs and during Mouse Craniofacial Development (A) ChIP-seq and ATAC-seq for PRS locus putative enhancer clusters EC1.45 (p300 Peak1 and Peak2), EC1.35 (S1 and S2), and EC1.25 (S3–S6). (B) Luciferase reporter assays for EC1.45, EC1.35 (S1 and S2) and EC1.25 (S3–S6) in hCNCCs (left) and hESCs (right). (C) Schematic outlining craniofacial domains at E9.5 and E11.5. BA1-2, branchial arch 1-2; FNP, frontonasal prominence; LNP, lateral nasal process; MdP, mandibular process; MNP, medial nasal process; MxP, maxillary process. (D) *In situ* hybridization (ISH) for *Sox9* at E9.5 and E11.5. (E) Mouse LacZ reporter assay for EC1.45, EC1.35 (S1 and S2), and EC1.25 (S3–S6 tested individually) at E9.5 and E11.5. (F) HREM for an EC1.45 LacZ reporter embryo at E11.5 (frontal view, top; parasagittal section, bottom). White arrow, activity in the MdP. See also Figure S3 and Tables S1 and S2.

Many large non-coding deletions identified in PRS patients map to the *SOX9* locus but are mostly non-overlapping, suggesting the presence of multiple regulatory elements with non-redundant functions whose loss leads to similar phenotypic outcomes (Amarillo et al., 2013; Benko et al., 2009; Gordon et al., 2014). Additionally, numerous translocation breakpoints have been identified that displace much of the distal *SOX9* gene desert away from the remainder of the locus (Figure 1A; Benko et al., 2009). To identify candidate hCNCC enhancer elements that map within regions of the *SOX9* gene desert lost in PRS patients, we used chromatin immunoprecipitation sequencing (ChIP-seq) and assay for transposase-accessible chromatin using sequencing (ATAC-seq) datasets from *in-vitro*-derived hCNCCs (Prescott et al., 2015; this study; Figure 1A). Among the candidate enhancers identified within the *SOX9* TAD, three enhancer clusters were located at the far centromeric end of the *SOX9* gene desert upstream of the PRS translocation breakpoint region and overlapped with at least one of the large deletions seen in patients with PRS. Each cluster contained two or more discrete binding peaks for the general coactivator p300, was enriched for the active enhancer marks H3K27ac and H3K4me1, and corresponded to regions of open chromatin (Prescott et al., 2015; Figures 1A and 2A; Calo and Wysocka, 2013). All three putative enhancer clusters were located over 1 Mb upstream of the *SOX9* gene (Figure 1A) and were named to reflect their genomic arrangement: enhancer cluster 1.45 (EC1.45) is 1.45 Mb upstream of *SOX9*, EC1.35 is 1.35 Mb upstream of *SOX9*, and EC1.25 is 1.25 Mb upstream of *SOX9* (Figures 1A and 2A).

Importantly, the three clusters of putative enhancers were not marked by active chromatin marks in human embryonic stem cells (hESCs) (Figure 1A) or other available profiled cell types (Figure S1A), except for human fetal craniofacial tissues (Figure S1B; Wilderman et al., 2018), suggesting that the putative enhancers exhibit cell-type-specific activity in the neural crest and developing face. Indeed, activation of these putative enhancers coincided with a strong increase in *SOX9* expression during transition from hESCs through neuroectodermal spheres (NECs) to hCNCCs (Figures S1C–S1E). Therefore, epigenomic signatures identified three putative hCNCC ECs overlapping sequences lost in PRS patients.

### PRS Region Candidate ECs Make Long-Range Contacts with the *SOX9* Promoter

To determine whether PRS region candidate ECs make contact with the *SOX9* promoter over more than a megabase of genomic space, we performed *SOX9* promoter-anchored Capture-C assays (Davies et al., 2016) in hESCs, NECs, early-migrating hCNCCs (hereafter called early hCNCCs), or late-passage hCNCCs (hereafter called late hCNCCs) (Prescott et al., 2015). In hESCs, the *SOX9* promoter formed contacts that spanned the previously defined TAD (Dixon et al., 2012, 2015), with the majority of interactions confined to the telomeric side, and also showed frequent interactions with CTCF (CCCTC-binding factor)/cohesin sites across the locus (Figures 1A, 1B, and S1F). A strong shift in interaction frequencies was apparent during hCNCC differentiation. In particular, extreme long-range interactions with the EC1.45, EC1.35, and EC1.25 putative ECs at the far centromeric end of the TAD substantially increased in late hCNCCs compared with hESCs (Figures 1B, S2A, and S2B), but the dynamics for each EC were distinct. Specifically, EC1.35 already contacted the *SOX9* promoter in hESCs (Figures S2A and S2B) and, notably, was occupied by CTCF and cohesin in hESCs and hCNCCs. This was mirrored by a similarly bound CTCF site 2 kb upstream of the *SOX9* promoter, suggesting that these genome-organizing proteins may facilitate a developmentally stable long-range interaction between the *SOX9* promoter and the distal region of the TAD (Arzate-Mejía et al., 2018; Guo et al., 2015; Ren et al., 2017; Schoenfelder and Fraser, 2019; de Wit et al., 2015). In comparison, EC1.25 and EC1.45 did not interact frequently with the *SOX9* promoter in hESCs by Capture-C (Figures S2A and S2B), and only in early and late hCNCCs did the contact frequency increase.

To confirm these cell-type-specific interactions, we performed reciprocal Capture-C experiments anchored at each of the three PRS region candidate ECs. Again, we observed an increase in contact frequency with the *SOX9* promoter in late hCNCCs compared with hESCs (Figure S2C). Importantly, Capture-C performed from other gene promoters in nearby TADs, including *KCNJ2*, *COG1*, and *SDK2*, did not reveal interaction with the PRS region putative ECs and did not cross the *SOX9* TAD boundaries (Figure S1F). The extreme long-range candidate enhancers at the PRS locus make selective contacts with the *SOX9* gene promoter in a disease-relevant cell type.

### Two PRS Locus Candidate ECs Drive Reporter Gene Expression in hCNCCs and in Developing Mouse Facial Structures

To investigate the regulatory potential of the putative PRS region ECs, we tested their capacity to activate transcription in a luciferase assay. We cloned the entire human EC1.45 region, including both p300 peaks, upstream of a luciferase reporter gene with an SV40 minimal promoter. Given their greater size, we combined the two p300 peaks for EC1.35 (S1 and S2) and the four p300 peaks for EC1.25 (S3–S6) (Figure 2A). EC1.45 and EC1.25 were found to be extremely strong drivers of transcription in hCNCCs, rivalling the activity of the viral SV40 enhancer positive control (Figure 2B, left panel). However, despite harboring epigenetic marks suggestive of active enhancer identity (albeit weaker than EC1.45 or EC1.25), EC1.35 was not active in the luciferase assay, suggesting that it is not a strong driver of transcription, at least in the examined context. As expected, none of the three ECs was active in hESCs (Figure 2B, right panel).

To characterize the spatiotemporal activity of the PRS locus enhancers during development, we utilized an *in vivo* LacZ enhancer reporter assay at two mouse embryonic stages, embryonic days 9.5 (E9.5) and E11.5 (Figure 2C). Human EC1.45 and a number of the constituent p300 peaks for human EC1.25 were active during mouse development, exhibiting reproducible activity patterns that mirrored distinct spatiotemporal subdomains of endogenous *SOX9* craniofacial expression (Figure 2D). This included activity in embryonic domains that will form the mandible: the first branchial arch at E9.5 (i.e., S3 and S5 of EC1.25) and the mandibular process at E11.5 (i.e., EC1.45 and S3 and S6 of EC1.25; Figures 2C and 2E and S3A–S3F; Tables S1 and S2). Mandibular activity of EC1.45 was further confirmed by high-resolution episcopic microscopy (HREM) (Figures 2F, S3G, and S3H). Similar to the *in vitro* luciferase assay, human EC1.35 did not display activity in the developing facial structures nor in any other tissues at either developmental stage (Figure 2E).

### Heterozygous Ablation of PRS Region ECs Causes an Allele-Specific Reduction in *SOX9* Expression

**Figure 3:**
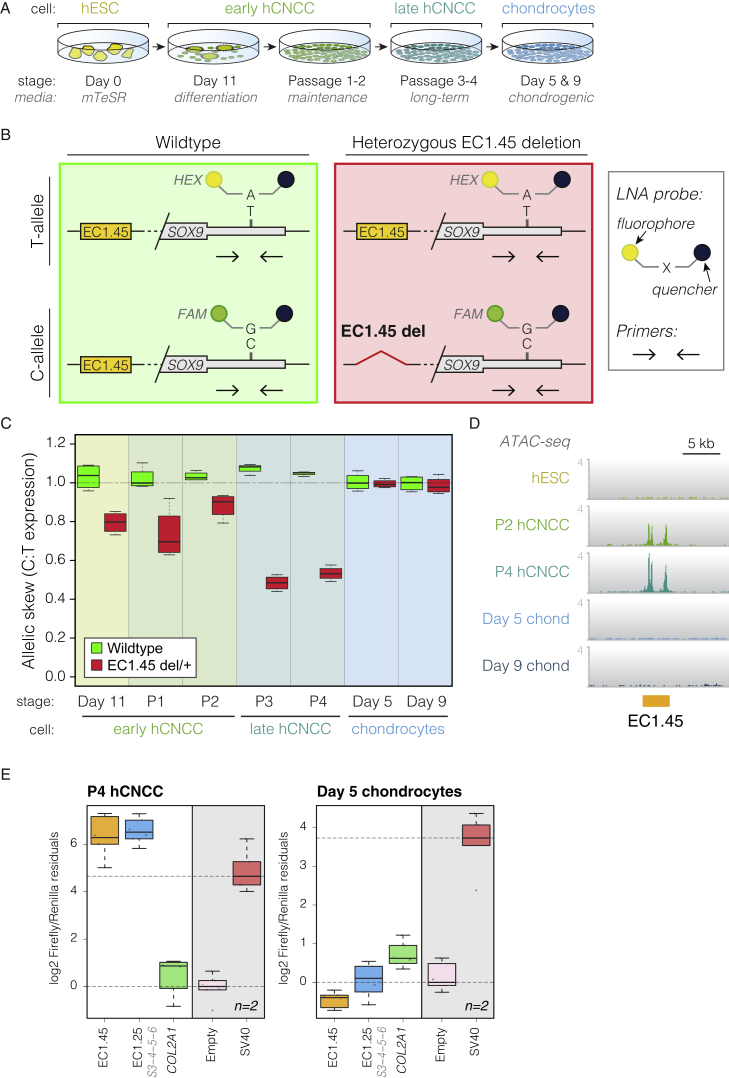
Heterozygous PRS Enhancer Deletion *In Vitro* Affects *SOX9* Expression during a Restricted Window of Development (A) Overview of differentiation, including early hCNCCs at day 11, passage 1–2 early hCNCCs, passage 3–4 late hCNCCs, and chondrocytes on days 5 and 9. (B) Schematic of allele-specific RT-ddPCR, indicating primers and LNA probes (HEX/FAM) for the T/C SNP (rs74999341) in the *SOX9* 3′ UTR. Shown are wild-type (left) and heterozygous EC1.45 deletion (right). (C) RT-ddPCR for wild-type (green boxplot) and EC1.45 heterozygous deletion (red), plotting *SOX9* C:T expression ratio. (D) ATAC-seq reveals hCNCC-specific accessibility for EC1.45. Shown are representative traces from 3–4 replicates. (E) Luciferase assay for late hCNCCs (left) and chondrocytes (right). A *COL2A1* enhancer is active in both cell types, whereas EC1.45 and EC1.25 become inactive in chondrocytes. See also Figure S4 and Table S3.

To directly characterize the contribution of the human EC1.45 and EC1.25 enhancers to *SOX9* gene regulation during hCNCC differentiation, we generated hESC lines with heterozygous deletions of EC1.45 or EC1.25 (Figures 3A and S4A–S4D; Ikeda et al., 2018). To determine the effect of these deletions on *SOX9* expression, we developed an allele-specific reverse transcriptase digital droplet PCR (RT-ddPCR) assay that distinguished a single-nucleotide polymorphism (SNP) in the 3′ UTR of the *SOX9* gene (T or C) (Figures 3B and S4E) and linked this in *cis* to the presence or absence of EC1.45 or EC1.25 via genome-wide phasing (Table S3).

During CNCC differentiation, the two alleles of *SOX9* were expressed at nearly equivalent levels in wild-type cells (Figures 3C and S4F, green boxplots) regardless of changes in overall *SOX9* expression (Figures S1C–S1E). In contrast, enhancer deletion was associated with a striking allelic skew in *SOX9* expression, indicating that loss of EC1.45 or EC1.25 disrupted normal regulation of *SOX9* (Figures 3C and S4F, red boxplots). The effect of EC1.45 enhancer deletion on *SOX9* expression was larger than that of EC1.25 deletion, especially in passage 3 (P3)–P4 late hCNCCs, where it led to 50%–55% lower expression of the mutated allele. This greater effect on *SOX9* expression in late hCNCCs was in keeping with an observed ~4.5-fold increase in EC1.45 activity between P2 and P4 hCNCCs (Figure S4G). In summary, EC1.45 and EC1.25 are required for normal expression of *SOX9* in the cranial neural crest, thus establishing some of the longest-range functional enhancer-gene interactions reported to date in the human genome.

### PRS Region Enhancers Are Decommissioned in Cranial Chondrocytes

SOX9 has two sequential critical roles in development of the mandible; first in specification and migration of CNCCs and second during chondrogenesis and formation of Meckel’s cartilage, the developmental precursor of the lower jaw (Amano et al., 2010; Wyganowska-Świa̧tkowska and Przystańska, 2011). We therefore tested whether EC1.45 and EC1.25 also regulate *SOX9* expression in cranial chondrocytes derived from hCNCCs (Figure 3A; differentiation validated in Figures S4H and S4I). Remarkably, cranial chondrocytes derived from hCNCCs heterozygous for EC1.45 or EC1.25 enhancer deletions did not show allelic skew in *SOX9* expression, indicating that the requirement for these enhancers in regulation of *SOX9* transcription is highly cell type restricted (Figures 3C and S4F). In agreement, ATAC-seq analysis revealed that EC1.45 and EC1.25 enhancers lost hypersensitivity during differentiation of hCNCCs to chondrocytes (Figures 3D and S4J), and luciferase reporter assays further confirmed that the regulatory potential of EC1.45 and EC1.25 was sharply reduced in chondrocytes (Figure 3E). Together, these data reveal that, despite high *SOX9* expression in chondrocytes, the PRS-associated enhancers have restricted and transient activity during CNCC development and become decommissioned during chondrogenesis, defining a developmental window for disease etiology.

### Two Short Segments Act Synergistically to Drive the Majority of EC1.45 Enhancer Activity

**Figure 4:**
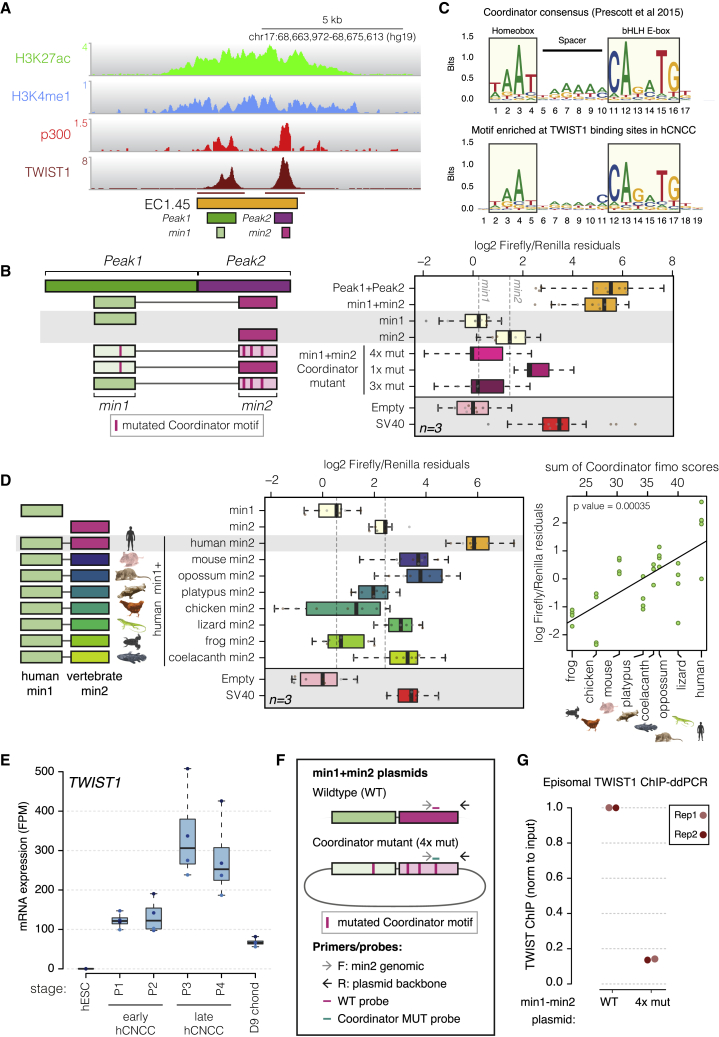
Dissection of EC1.45 Enhancer Region Uncovers a Core Role of the Coordinator Motif and TWIST1 Binding in Developmental Enhancer Regulation (A) TWIST1 ChIP-seq peaks (marked under track) at EC1.45 overlap p300 Peak1 and Peak2 and minimally active sequences (min1 and min2). (B) Luciferase assay for EC1.45 min1 and min2, tested separately and combined, along with Coordinator mutant sequences. Left: schematic of the constructs. (C) Coordinator motif (top; Prescott et al., 2015) compared with the motif enriched at TWIST1 binding sites in hCNCCs (bottom). (D) Luciferase assay for the heterologous enhancer sequence for human min1 plus vertebrate min2. Left: schematic of the constructs. A scatterplot depicts the luciferase signal compared with the sum of Coordinator scores (ANOVA p = 0.00035; right). (E) *TWIST1* is upregulated during hCNCC differentiation and reduced in chondrocytes (fragments per million [FPM]). (F) Schematic of plasmids, primers, and probes for ChIP-ddPCR for wild-type (WT) and Coordinator mutant (4x mut) min1+min2 plasmids. F, forward; R, reverse. (G) TWIST1 ChIP-ddPCR for P4 late hCNCCs transfected with the plasmids in (F), normalized to input, and WT adjusted to 1. Two biological replicates are depicted. See also Figure S5.

To interrogate sequence features critical for hCNCC-specific activity of the PRS ECs, we focused on EC1.45, whose deletion is associated with greater allelic imbalance in *SOX9* expression in hCNCCs. First, we tested the enhancer activity of the two constituent EC1.45 p300 peaks (Peak1 and Peak2; Figure 4A) in luciferase reporter assays. Intriguingly, individually, the two p300 peaks exhibited only weak enhancer activity, whereas Peak1+Peak2 led to activation greater than the sum of the two regions, indicative of synergistic activity (Figures S5A–S5C). To further refine regions of enhancer activity within EC1.45, we performed a tiling deletion screen across Peak1+Peak2 (Figure S5A) and identified two minimal regions (overlapping deletions 3–4 in Peak1 and deletions 10–11 in Peak2) whose loss lead to a significant reduction in luciferase reporter activity, min1 and min2, respectively. Importantly, min1+min2 recapitulated the activity and synergy of Peak1+Peak2 and accounted for nearly the full activity of EC1.45 (Figures 4B and S5B). Of interest, three of the constituent putative enhancers from EC1.25 also act independently as enhancers in late hCNCCs, whereas combination of all four individual elements appears to similarly drive synergistic activation of luciferase expression (Figure S5C). Unsurprisingly, the two constituent EC1.35 p300 peaks were not active enhancers by luciferase assay (Figure S5C). Therefore, we identify two core enhancer elements within EC1.45 (and three within EC1.25) that are weak enhancers individually but work together in a robustly synergistic manner to activate gene expression.

### Coordinator Motifs Are Essential for Activity and Synergy of EC1.45 Enhancers

In our previous study investigating sequence features associated with divergence of enhancer activity between human and chimpanzee CNCCs, we identified a long bipartite sequence that we called “Coordinator” (Prescott et al., 2015; Figure 4C, top). Of all motifs tested, Coordinator had the greatest effect on surrounding chromatin features and affected the highest number of enhancers (Prescott et al., 2015), suggesting a privileged role in establishment of enhancer competence in CNCCs. Strikingly, there are seven Coordinator motifs within the EC1.45 Peak1+Peak2 region, four of which fall within min1+min2 (Figure S5D). Mutations of all four motifs in min1+min2 diminished activity to the level of the empty vector, while mutation of the Coordinator sequence in min1 brought the activity of min1+min2 down to a level similar to min2 alone (Figure 4B). Similarly, mutation of the three Coordinator sequences in min2 brought the activity of min1+min2 down to a level similar to min1 alone (Figure 4B), indicating that the Coordinator motif is essential for the activity and synergistic function of the EC1.45 enhancers. A mutation screen of each of the seven motifs within the Peak1+Peak2 region further supported that the most substantial contribution of Coordinator motifs to overall enhancer activity is within the min1 and min2 regions and revealed that mutation of all seven Coordinator motifs led to a reduction of activity below the baseline level of the minimal promoter control (p = 0.027). Notably, this suggests that repressive sequence features exist within the enhancer region that are unmasked by loss of Coordinator sites (Figure S5E) and may be harbored within the del1-del2 region (Figure S5A; p < 0.0063).

### Coordinator Motif Content in the Deeply Conserved Region of EC1.45 Correlates with Enhancer Activity across Species

Interestingly, EC1.45 min2 is conserved at the sequence level from human to the lobe-finned fish coelacanth across ~400 million years of evolution (Figures S5F and S5G). To examine the relationship between the Coordinator motif content (estimated from Fimo; Figure S5H; Grant et al., 2011) of orthologous min2 regions and their enhancer activity, we cloned the min2 sequences from mouse, opossum, platypus, chicken, lizard, frog, and coelacanth downstream of the human min1 sequence and assessed their combined activity by luciferase assay (Figure 4D). Strikingly, an increased Coordinator score was associated with increased enhancer activity (Figure 4D, right panel). These changes in activity did not simply recapitulate the phylogenetic relationship between the examined species because, for example, the most distantly related coelacanth sequence was relatively high in Coordinator content and enhancer activity, suggesting that the presence of the Coordinator motif rather than merely evolutionary drift drive the observed changes in activity.

### TWIST1 Regulates EC1.45 Enhancers in a Coordinator-Dependent Manner

We next sought to uncover the *trans*-regulatory inputs that control EC1.45 activity. The Coordinator sequence resembles an E-box- and Homeobox-like motif, separated by 6 bp, although the factors that bind are so far unknown. E-box motifs are recognized by basic helix-loop-helix (bHLH) transcription factors, and we noted that *TWIST1* was among the most highly expressed bHLH factors in hCNCCs. *TWIST1* levels tightly coincided with EC1.45 enhancer activity, being strongly upregulated during hCNCC differentiation (with highest expression in late hCNCCs) and downregulated during chondrogenesis (Figure 4E; compare with to EC1.45 enhancer activity in Figure 3C). TWIST1 is also known to play an essential role in neural crest biology and craniofacial development (Bildsoe et al., 2009; Parsons et al., 2014; Qin et al., 2012). Furthermore, *Twist1* inactivation in NCCs populating the mandibular arch in mice leads to micrognathia and cleft palate (Zhang et al., 2012), phenotypes overlapping those seen in PRS.

Genome-wide analysis of TWIST1 ChIP-seq performed in hCNCCs revealed that the top enriched sequence matched the Coordinator motif (Figure 4C, bottom), followed by a canonical TWIST1 E-box motif (Figure S5I), suggesting that, in hCNCCs, a substantial fraction of TWIST1 chromatin binding occurs in the context of the Coordinator motif. In keeping with the presence of multiple Coordinator motifs, TWIST1 binds to both EC1.45 constituent enhancers in hCNCCs (Figure 4A). To assess whether this binding is dependent on the Coordinator sequence, we developed an episomal TWIST1 ChIP-ddPCR assay that distinguished transfected plasmids containing wild-type or Coordinator mutant min1+min2 sequences (Figure 4F). In this assay, the strong TWIST1 binding observed for the wild-type min1+min2 sequence was greatly diminished by mutation of the Coordinator sequences (Figure 4G). These results establish that TWIST1 binds to the min1 and min2 regulatory sequences in CNCCs in a Coordinator motif-dependent manner.

### Mouse Mandibular Development Is Highly Sensitive to Changes in *Sox9* Gene Dosage

**Figure 5:**
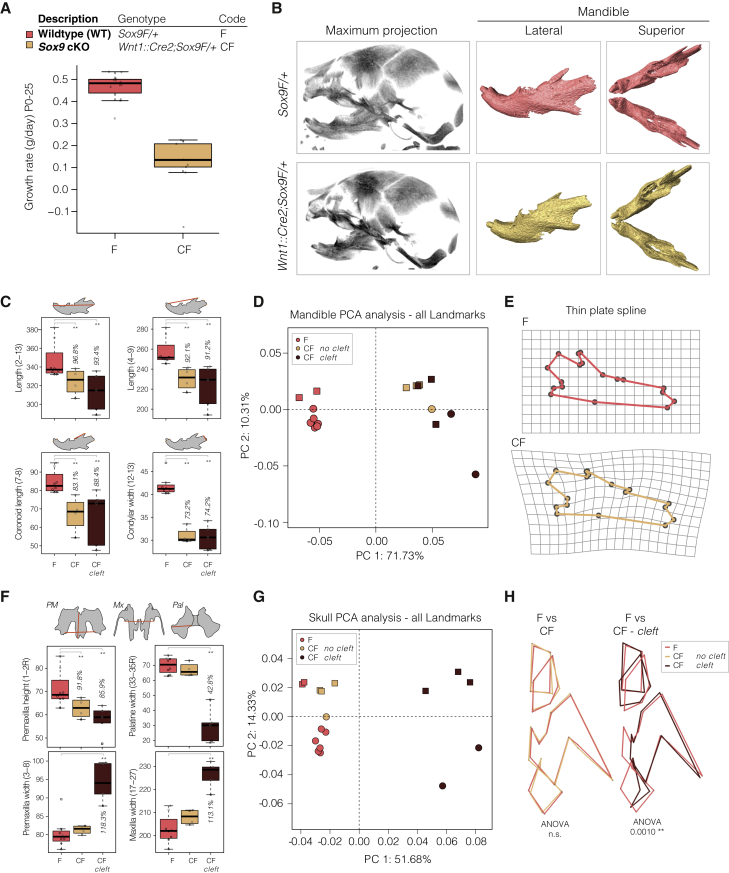
Conditional Neural Crest-Specific *Sox9* Heterozygous Mutant Embryos Have Craniofacial Defects and Fail to Thrive in the Neonatal Period (A) Boxplot of postnatal growth rate in grams per day for mutant *Wnt1::Cre2;Sox9F/+* (CF) and WT *Sox9F/+* (F) pups (ANOVA p = 1.793e−07). (B) MicroCT scans of E18.5 WT (top) and mutant (bottom) embryos, maximum intensity projection (left), and segmented hemimandibles (right). (C) Boxplot of distance measurements for WT (F) and mutant mandibles with (CF cleft) and without (CF) cleft palate. Data are from two litters (17 embryos). Statistical test: ANOVA. (D) PCA of mandible landmarks following Procrustes analysis. Mutant (CF) and WT (F) mandibles are separated by PC1 regardless of clefting. (E) Morphometric landmarks for WT (top, F) and mutant (bottom, CF) mandibles projected onto a thin plate spline. All 18 landmarks differed significantly between WT and mutant mandibles by Hotelling test (p < 0.0006). (F) Boxplot of distance measurements for WT (F) and mutant midfacial elements with (CF cleft) and without (CF) cleft palate. PM, premaxilla; Mx, maxilla; Pal, palatine bones. Statistical test: ANOVA. (G) PCA of skull landmarks following Procrustes analysis. Mutant skulls without cleft (CF) cluster with wild-type skulls (F). (H) Wireframe outline of nasal bone, PM, Mx and Pal for half a skull for WT (F, dark pink) and mutant skulls without cleft (CF, yellow, left) or with cleft (CF cleft, brown, right). For PCA, different shape markers represent independent litters. See also Figure S6 and Table S4.

With the identification of two ECs at the PRS locus that regulate *SOX9* gene dosage in hCNCCs, we next turned to mouse models to probe the morphological effect of *Sox9* dosage perturbation on craniofacial development. Previous work showed that heterozygous deletion of *Sox9* recapitulates many aspects of campomelic dysplasia (Bi et al., 2001). To characterize the effect of neural crest-specific *Sox9* haploinsufficiency, we crossed mice carrying a floxed (F) *Sox9* allele (Akiyama et al., 2002) with mice carrying the second-generation *Wnt1::Cre2* driver (C) that directs *Cre* expression in the neural crest just before or during delamination from the neural tube (Lewis et al., 2013; Figure S6A). Many heterozygous *Wnt1::Cre2;Sox9F/+* (CF; Cre Flox) animals died in the neonatal period from postnatal day P0–P12 or failed to gain weight at the same rate as their wild-type siblings (Figures 5A and S6B). To investigate the cause of neonatal lethality and reduced fitness, we performed micro-computed tomography (microCT) to monitor craniofacial skeletal development at E18.5 (Figure 5B). Clefting was detected in the maxilla and palatine bones in 50% of mutant embryos, a phenotype in PRS patients thought to be a secondary consequence of mandibular hypoplasia (Tan and Farlie, 2013). Although this link remains to be established in the mouse, the observed cleft palate is most likely the cause of postnatal lethality because of feeding or breathing difficulties (Tan and Farlie, 2013).

To further quantify craniofacial defects, we performed morphometric landmarking (Ho et al., 2015; Welsh et al., 2018), focusing first on the mandible because micrognathia is a diagnostic characteristic of PRS as well as a feature of campomelic dysplasia (Foster, 1996; Robin, 1994; Tan and Farlie, 2013; Figures S6C and S6D). From this analysis, we quantified a reduction in mandible length for *Wnt1::Cre2;Sox9F/+* embryos and gross changes in the shape of the ramus, including a dramatically hypoplastic coronoid process and reduced condylar process width, recapitulating aspects of human patient phenotypes with *SOX9* haploinsufficiency (Figure 5C). Notably, these differences in mandibular shape and size were fully penetrant in all embryos analyzed, regardless of the presence of cleft palate, as illustrated by principal-component analysis (PCA) based on calculated Procrustes distance (Figures 5C and 5D). The dramatic changes in mandibular morphology can be illustrated by projecting mandibular landmarks onto a thin plate spline (Figure 5E).

We next analyzed the remaining skull morphology (Figures S6E–S6G), and interestingly, although *Wnt1::Cre2;Sox9F/+* mutant embryos with a cleft palate displayed a number of measurable skull anomalies, we did not detect significant alterations in skull length or width or midfacial length in mutant embryos without a cleft (Figures 5F and S6H). Indeed, PCA revealed that skull shapes of non-clefted *Wnt1::Cre2;Sox9F/+* animals cluster with those of the wild-type embryos and away from the clefted heterozygotes (Figure 5G), with no significant change in overall skull shape (Figure 5H). Therefore, despite the broad expression and function of *Sox9* throughout developing craniofacial structures, the mandible exhibits heightened and fully penetrant sensitivity to a 50% reduction of *Sox9* gene dosage during mouse neural crest development compared with other craniofacial structures where phenotypes are of variable expressivity.

### The Mouse Orthologous EC1.45 Sequence Exhibits Conserved Spatiotemporal Activity Pattern but Weakened Contribution to *Sox9* Expression Relative to Human EC1.45

**Figure 6:**
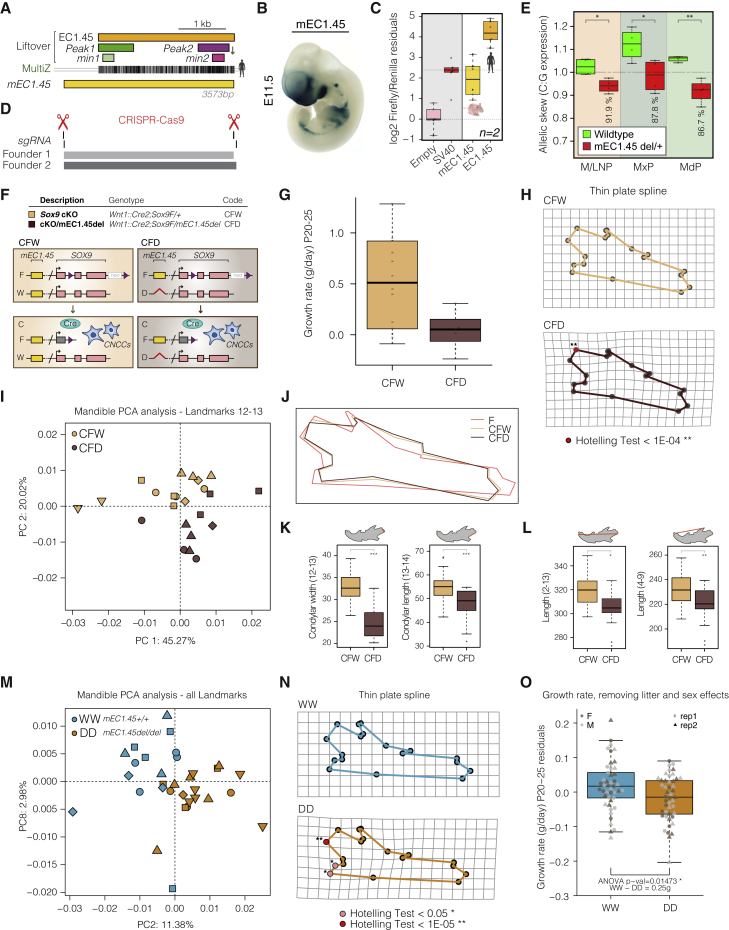
Reduction in *Sox9* Activity Affects Mouse Craniofacial Development in a Dose-Dependent Manner (A) Schematic of mouse orthologous mEC1.45 with liftover of human EC1.45, Peak1, Peak2, min1, and min2 sequences and human-to-mouse MultiZ alignment. (B) Mouse LacZ reporter assay for mEC1.45 at E11.5. (C) Luciferase assay for human EC1.45 and mouse mEC1.45. (D) Location of single guide RNAs (sgRNAs) and founder 1 and 2 mEC1.45 deletions (aligned with A). (E) RT-ddPCR for *Sox9* from WT and mEC1.45del/+ dissected E11.5 craniofacial tissues, plotted as C:G allelic ratio, mEC1.45 deleted on the C allele. t-test:^∗^p < 0.05, ^∗∗^p < 0.01. (F) Schematic of *Sox9* heterozygous conditional knockout *Wnt1::Cre2;Sox9F/+* (CFW) and compound heterozygous *Wnt1::Cre2;Sox9F/mE*C*1.45del* (CFD) mice with *Sox9* deleted in CNCCs on one allele and mEC1.45 deleted on the other. Purple triangles, loxP sites; neo, neomycin resistance. (G) Boxplot of postnatal growth rate (P20–P25, grams per day) for CFW and CFD animals. ANOVA p = 0.01676. (H) Landmarks for CFW (top) and CFD (bottom) mandibles projected onto a thin plate spline. Landmarks that differ significantly by Hotelling test are highlighted in red (p < 1E−04). (I) PCA plot of mandible landmarks 12 and 13 following Procrustes analysis at E18.5 for 5 litters (23 embryos) of CFW (yellow) and CFD (brown) embryos. (J) Procrustes-transformed average mandible wireframes for WT (dark pink, FW), CFW (yellow), and CFD (brown) embryos. (K) Measurements of width and length of the condylar process for CFW (yellow) and CFD (brown) mandibles. For condylar width, ANOVA p = 1.52E−07. For condylar length, ANOVA p = 1.11E−04. (L) As for (K); two measurements of mandible length; 2–13, ANOVA p = 0.00143; 4–9, ANOVA p = 0.00687. (M) PCA plot of all mandible landmarks following Procrustes analysis for WT (*mE*C*1.45+/+*, blue, WW) and homozygous mutant (*mE*C*1.45del/del*, orange, DD) embryos at E18.5 for 5 litters (32 embryos). (N) Landmarks for WW and DD mandibles projected onto a thin plate spline. Landmarks that differ significantly by Hotelling test are highlighted in pink (p < 0.05) and red (p < 1E−05). (O) Boxplot of postnatal growth rate (P20–P25, g/day) for WW and DD embryos. Two replicate groups plotted as residuals of linear regression; ANOVA p = 0.01473. For PCA plots, different shape markers represent independent litters. See also Figure S7 and Tables S1 and S2.

To assess whether the spatiotemporal activity of the EC1.45 and EC1.25 elements was conserved for the orthologous mouse sequences (located 1.21 Mb and 1.04 Mb from the mouse *Sox9* promoter, respectively; for clarity, we refer to these regions as mEC1.45 and mEC1.25), we again utilized *in vivo* LacZ reporter assays. Similar to the human sequence, mEC1.45 was active in the frontonasal prominence at E9.5 and also in the maxillary and mandibular processes and limb buds at E11.5 (Figures 6A, 6B, S7A, and S7B). Of note, a sub-region of this sequence was tested previously in the VISTA Enhancer Browser (mm628) (Visel et al., 2007; Figures S7A and S7C). In contrast, for the three human EC1.25 constituent enhancers with craniofacial activity (Figure 2E), there was no reproducible activity for mouse orthologous S3 and S5 enhancers and reduced craniofacial activity for the S6 ortholog at E11.5 in a domain not including precursors of the mandible (Figure S7D). To compare the activity of human EC1.45 and its mouse ortholog in a more quantitative assay, we performed parallel luciferase assays in hCNCCs. Although mEC1.45 is indeed an active enhancer in hCNCCs, it is a much less potent activator of luciferase expression than the human ortholog (around 15-fold lower), indicating a divergence in enhancer strength (Figure 6C), consistent with reduced Coordinator content for mouse min2 (Figures 4D and S5H).

Based on the conserved, albeit weak, activity of mEC1.45, we performed pronuclear injection of CRISPR-Cas9 ribonucleoprotein (RNP) complexes to target the region for deletion (Figure 6D) and established two distinct founder lines (Figures 6D, S7E, and S7F). To determine the effect of mEC1.45 deletion on *Sox9* expression, we crossed *mEC1.45del/+* FVB mice with wild-type C57BL/6J mice, dissected craniofacial processes from embryos at E11.5 and performed allele-specific RT-ddPCR for *Sox9* utilizing strain-specific SNPs (Figure S7G). Analysis of *Sox9* expression in the combined medial nasal process (MNP) and lateral nasal process (LNP), maxillary process (MxP), and mandibular process (MdP) revealed that in wild-type embryos, *Sox9* was expressed at similar levels from the FVB and C57BL/6J alleles (Figure 6E, green boxplots). In contrast, in *mEC1.45del/+* embryos, *Sox9* expression was significantly reduced for the FVB allele carrying the enhancer deletion, with the greatest reduction observed for the MdP (Figure 6E, red boxplots; p < 0.032). Consistent with the weaker activity of mEC1.45 compared with human, deletion of mEC1.45 caused quantitatively milder (8% in the MNP/LNP, 12% in the MxP, and 13% in the MdP) reduction in *Sox9* expression from the mutant allele compared with a much greater reduction of *SOX9* expression in EC1.45del/+ late hCNCCs (~50%–55%). At an earlier stage of development, E9.5, we also observed a modest reduction in *Sox9* expression from the mEC1.45del mutant allele, with the most significant effect seen for the frontonasal prominence (FNP), consistent with the enhancer activity pattern at this stage (Figures S7B and S7H). Therefore, although the spatiotemporal activity of mEC1.45 is conserved, there is a substantially diminished strength of activity and input into *Sox9* expression compared with the human sequence.

### Deletion of Mouse Orthologous EC1.45 Affects Mandible Morphology and Exacerbates PRS-like Phenotypes Associated with *Sox9* Heterozygosity

Considering the overall weaker regulatory activity of mEC1.45 compared with human, we chose a sensitized background strategy to first assess a possible function of mEC1.45 in craniofacial development. We therefore crossed *Wnt1::Cre2;mEC1.45del/+* females and *Sox9F/F* males and compared *Wnt1::Cre2;Sox9F/mEC1.45del* compound heterozygous mice (CFD [Cre Sox9-Flox Delete-mEC1.45]), for which all *Sox9* transcripts in CNCCs and derivatives are expressed from the allele with mEC1.45 deleted (Figure 6F) to *Wnt1::Cre2;Sox9F/+* conditional *Sox9* knockout animals (CFW [Cre Sox9-Flox Wild-type-mEC1.45]), for which the remaining allele expressing *Sox9* is wild-type. In this sensitized setting, we may predict exacerbated phenotypes compared with those seen in conditional *Sox9* heterozygotes.

We initially weighed surviving pups up to weaning and observed a decreased growth rate for compound mutant (CFD) animals compared with *Sox9* heterozygous (CFW) animals (Figure 6G). We next performed microCT and landmarking for E18.5 mandibles from the same cross, and Procrustes analysis followed by a Hotelling test revealed a landmark at the condylar process as most morphometrically distinct between genotypes (p < 1e–04, Figure 6H). Indeed, PCA analysis using landmarks at the condylar process clearly separated mandibular morphology for CFW and CFD embryos (Figure 6I). These results show that additional loss of the mEC1.45 enhancer exacerbates the changes in mandible morphology observed in the conditional heterozygous *Sox9* mutant (Figures 5E and 6J). Furthermore, quantification of condylar process length and width revealed a reduction for CFD compared with CFW embryos (p < 1.1e−4, ANOVA; Figure 6K), whereas overall mandible length was also reduced by 3%–5% (p < 0.007, ANOVA; Figure 6L). Therefore, ablation of a developmental EC that intersects a human disease locus exacerbates PRS-like phenotypes in a sensitized genetic background.

To determine whether mEC1.45 enhancer deletion alone, which, even in a homozygous setting, is expected to cause only a 13% reduction in *Sox9* expression (Figure 6E), results in altered jaw morphology, we performed microCT analysis for E18.5 embryos obtained from a cross between heterozygous *mEC1.45del/+* animals. Using all 18 mandibular landmarks, we were able to separate the wild-type (WW) and mEC1.45 homozygous knockout (DD) embryos by PCA, indicating a reproducible phenotypic alteration of mandibular shape when the mEC1.45 enhancer is ablated (Figure 6M). A Hotelling test again revealed that the ramus was the mandibular structure most affected by changes in *Sox9* dosage (Figure 6N). Although milder, these alterations in mandibular ramus morphology are reminiscent of phenotypes observed in PRS patients, as quantified by a number of studies (Bienstock et al., 2016; Chen et al., 2015; Chung et al., 2012; Suri et al., 2010; Susarla et al., 2017; Volk et al., 2020; Zellner et al., 2017; Table S4; Figure S6C). Finally, to address whether enhancer knockout results in failure to thrive, we weighed pups up to weaning age (P20–P25) and detected a reduction in weight gain for mEC1.45 knockout animals (Figure 6O). Collectively, these data show that even a subtle reduction in gene dosage, caused by enhancer loss, can lead to alterations of craniofacial morphology and result in reduced ability of an organism to thrive.

## Discussion

**Figure 7:**
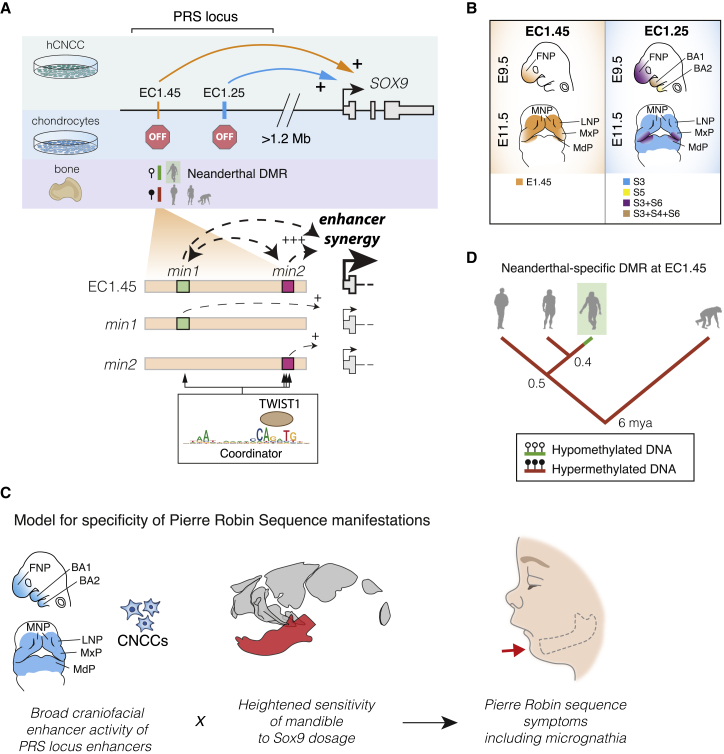
Summary of PRS Locus Enhancer Activity with a Proposed Model for PRS Etiology and Associated Neanderthal Differentially Methylated Region (DMR) Evolution (A) A model of EC1.45 and EC1.25 hCNCC-specific regulation of *SOX9* expression at extremely long distance followed by decommissioning in chondrocytes. A Neanderthal-specific hypomethylated region (HMR) overlaps EC1.45. Two minimal elements in EC1.45 have synergistic activity; i.e., (min1+min2) > (min1)+(min2). Coordinator motifs in min1 and min2 sequences are central for their activity and are bound by TWIST1. (B) EC1.45 and EC1.25 are active in the developing face. (C) A model for PRS etiology where by two features converge to confine disease phenotypes to the lower jaw. (D) Phylogenetic tree of the inferred regulatory evolution for an EC1.45 Neanderthal-specific hypomethylated region (HMR, green). From left to right: anatomically modern humans (AMHs), Denisovans, Neanderthals, and chimpanzees. mya, million years ago. See also Figure S6.

Given the phenotypic overlap between craniofacial abnormalities of campomelic dysplasia and PRS, it had been long speculated, but not formally demonstrated, that regulatory elements harbored by the PRS region deletions might regulate *SOX9* (Amarillo et al., 2013; Benko et al., 2009; Gordon et al., 2009, 2014). Furthermore, several distinct hypotheses have been put forth regarding the cellular origins of the disease (Tan et al., 2013). In this study, we shed light onto these long-standing questions, identifying and characterizing two clusters of enhancers 1.25 and 1.45 Mb upstream of the *SOX9* gene that fall within the PRS locus, are active during craniofacial development, make long-range contacts with the *SOX9* promoter, and dynamically regulate its expression during cranial neural crest development (Figures 7A and 7B). Importantly, these enhancers become inactive following hCNCC differentiation to chondrocytes, defining a developmental window for the etiology of craniofacial phenotypes observed in PRS (Figure 7A).

In some enhanceropathies, a number of patient-specific mutations overlap to reveal a single minimal element that is disrupted in the disorder (e.g. Gonen et al., 2018; Kortüm et al., 2011). In contrast, many of the PRS deletions described to date are non-overlapping and harbor one or the other EC identified here (Amarillo et al., 2013; Benko et al., 2009; Gordon et al., 2014). Interestingly, PRS patients with translocation breakpoints, in which both EC1.25 and EC1.45 are lost, appear to display more severe phenotypes (Benko et al., 2009). This suggests that loss of distinct enhancers that quantitatively affect *SOX9* dosage in CNCCs can lead to similar disease outcomes, whereas loss of the broader regulatory region via chromosomal translocation can have an additive effect on both *SOX9* gene dosage and lower jaw morphology. Indeed, our mouse modeling revealed that lower jaw development is sensitive to even small perturbations in *Sox9* gene dosage, with a range of phenotypes of increasing severity observed over a range of reductions in *Sox9* expression (Figures 5 and 6).

In our analysis of the 1.45 Mb EC, the two constituent p300-binding regions within EC1.45 are individually weak enhancers but display a striking combinatorial synergy far greater than the sum of the individual activities (Figure 7A). Previous studies looking at the relationship between multiple enhancers within super ECs have supported additive or redundant rather than synergistic activity of the constituent enhancer elements (Dukler et al., 2016; Hay et al., 2016; Moorthy et al., 2017; Shin et al., 2016).

The regulatory elements identified in our study represent the longest-range developmental enhancers involved in congenital malformations that have been described to date, at a distance of nearly 1.5 Mb from the regulated gene promoter. These enhancers provide a valuable paradigm for continuing investigation of long-range developmental gene regulation and its perturbation in human disease, and they join a small class of documented extreme long-range regulatory sequences that activate transcription at a more than 1-Mb genomic interval, such as the *Shh* ZRS and the *Myc* BENC and MNE enhancers (e.g., Bahr et al., 2018; Herranz et al., 2014; Lettice et al., 2003; Uslu et al., 2014). The enormous genomic distance begs questions about how the PRS-associated ECs can communicate with the *SOX9* promoter to drive tissue-specific regulation in a precise and robust manner. Interestingly, one of the PRS-associated candidate ECs, EC1.35, which does not harbor activity in reporter assays, contains a constitutive CTCF binding site and interacts with the *SOX9* promoter already in hESCs. This interaction is further augmented in hCNCCs, along with contacts between all three PRS-associated enhancers and between EC1.45 and EC1.25 and the *SOX9* promoter. Remarkably, a recent study where the centromeric *Sox9* TAD boundary was deleted in mice showed no significant effect on *Sox9* expression or examined phenotypes (Despang et al., 2019), suggesting that TAD integrity may not be required for these long-range interactions and *Sox9* regulation. However, although the EC1.35 element may not act as a canonical enhancer, it may instead participate in organizing extreme long-range contacts at the *SOX9* locus via formation of CTCF-cohesin-mediated chromatin loops.

When attempting to model human non-coding mutations at the orthologous PRS locus in mice, there were a number of challenges to consider. First, because of extensive reshaping of mammalian genomes during evolution by transposons and other genomic forces, many functional human enhancer regions do not have orthologous sequences in mice (Chuong et al., 2017; Villar et al., 2015; Yue et al., 2014). Second, even when the orthologous sequence exists, its regulatory activity may not be conserved or can differ in strength or relative contribution to the target gene dosage (Denas et al., 2015; Shen et al., 2012). This second challenge is well illustrated by EC1.45; although the orthologous sequence is present in mice and its spatiotemporal activity is conserved, mEC1.45 is a substantially weaker enhancer compared with its human counterpart, perhaps compensated for by additional mouse-specific CNCC enhancers at the locus (Figure S7I). Consequently, deletion of mEC1.45 results in only an ~6%–13% decrease in *Sox9* expression level compared with the 50%–55% *SOX9* reduction seen for human EC1.45 deletion. Nonetheless, it is quite remarkable that even such a slight reduction in *Sox9* gene dosage results in measurable changes in lower jaw shape and reduction in postnatal growth.

Despite the caveats outlined above, from our combined human and mouse results we can propose a model for the specificity of PRS manifestations where mutations at the far end of the *SOX9* gene desert perturb broadly active craniofacial developmental ECs and affect *SOX9* expression across the cranial neural crest. However, the heightened sensitivity of the mandible to *SOX9* gene dosage further restricts the manifestations to micrognathia, which can, in sequence, lead to additional PRS-associated phenotypes (Figure 7C). Our work raises the interesting question of why the mandible is more sensitive to *Sox9* dosage perturbation despite the broad expression of *Sox9* across craniofacial structures. We suggest two potential hypotheses. In the first, we note that distinct transcription factors and signaling components are expressed in the future upper and lower jaw during development; for example, high levels of *Hand2* and *Dlx5/6* are expressed in the mandibular but not MxP (Beverdam et al., 2002; Funato et al., 2016). Loss of this patterning through ablation of the upstream Edn1/Ednra signaling pathway leads to a striking jaw transformation (Minoux and Rijli, 2010). Therefore, if spatially restricted morpho-regulatory programs such as these are differentially sensitive to *Sox9* activity, then this could lead to tissue-selective effects on craniofacial development. An alternate hypothesis for the observed mandibular sensitivity to *Sox9* perturbation could be related to the differences in the trajectory of craniofacial skeletal development. In a somewhat atypical process, formation of the mandible is intimately associated with a cartilage “template” called Meckel’s, whereas, in contrast, the midfacial skeleton forms strictly via intramembranous ossification independent of any cartilage precursor. Therefore, should perturbation of *Sox9* expression in CNCCs affect the propensity or ability to differentiate into chondrocytes, it could account for the selective effect on mandibular development.

From an evolutionary standpoint, the mandible is extremely interesting because it is has widely divergent forms related to feeding and predation (Albertson and Kocher, 2006; Martinez et al., 2018). Furthermore, mandible shape evolution in hominins appears to be exceptionally rapid compared with any other primate clade (Raia et al., 2018) and includes shape changes within the ramus, including the condylar and coronoid processes and gonial angle—structures that are especially sensitive to slight alterations in *Sox9* expression in our mouse models (Meloro et al., 2015; Terhune et al., 2014, 2018). It is therefore tempting to speculate that some of this morphological divergence could be mediated by regulatory changes leading to minor differences in *SOX9* expression levels during CNCC development. In fact, EC1.45, featured in this study, overlaps a Neanderthal-specific hypomethylated region from bone samples (based on reconstructed DNA methylation maps; Gokhman et al., 2014, 2020; Figures 7A, 7D, and S6I). Although somewhat speculative, this suggests that the Neanderthal enhancer element might have retained regulatory activity longer during development (because DNA methylation is generally associated with silencing) compared with the human enhancer, which becomes decommissioned during chondrogenesis and is hypermethylated in human bones of various origins (Figures 3C–3E). Together, the PRS locus enhancers represent a fascinating locus for future investigation of extreme long-range gene regulation in development and disease, and across evolutionary time.

### Limitations of Study

As outlined above, there are a number of challenges and limitations when attempting to model a human enhanceropathy in mice because of remodeling of the enhancer landscape across evolutionary time. In our study, this is exemplified by the weakened enhancer activity of mE1.45 compared with the human counterpart, and associated lower contribution to *Sox9* expression. An additional limitation relates to the mouse strains used in the study; *Wnt1::Cre* and *Sox9F/F* mice are on an C57BL/6J background, whereas *mEC1.45* deletion was generated on the FVB background. These different genetic backgrounds may cause a differential sensitivity to *Sox9* perturbation because of other modifying variants in the genome.

## STAR★Methods

### Key Resources Table

**Table:** 

REAGENT or RESOURCE	SOURCE	IDENTIFIER
**Antibodies**		

Rabbit polyclonal p300 (discontinued) – ChIP	Santa Cruz Biotechnology	Cat# sc-585; RRID: AB_2231120
Rabbit polyclonal H3K4me1 – ChIP	Abcam	Cat# ab8895; RRID: AB_306847
Rabbit polyclonal H3K27ac – ChIP	Active Motif	Cat# 39133; RRID: AB_2561016
Rabbit polyclonal H3K4me3 – ChIP	Active Motif	Cat# 39159; RRID: AB_2615077
Rabbit polyclonal CTCF – ChIP	Cell Signaling	Cat# 2899; RRID: AB_2086794
Rabbit polyclonal RAD21 – ChIP	Abcam	Cat# ab992; RRID: AB_2176601
Mouse monoclonal TWIST1 – ChIP	Abcam	Cat# ab50887; RRID: AB_883294

**Chemicals, Peptides, and Recombinant Proteins**		

mTeSR	Stem Cell Technologies	Cat# 85850
Matrigel Growth Factor Reduced (GFR) Basement Membrane Matrix	Corning	Cat# 356231
ReLeSR	Stem Cell Technologies	Cat# 05872
Collagenase IV	GIBCO	Cat# 17104019
DMEM/F12 1:1 medium, with L-glutamine; without HEPES	GE Healthcare	Cat# SH30271.FS
Neurobasal Medium	Thermo Fisher Scientific	Cat# 21103049
Gem21 NeuroPlex Supplement With Vitamin A	Gemini Bio-Products	Cat# 400-160
N2 NeuroPlex Supplement	Gemini Bio-Products	Cat# 400-163
Antibiotic-Antimycotic (100X)	GIBCO	Cat# 15240062
GlutaMAX Supplement (100X)	Life Technologies	Cat# 35050061
Recombinant Human FGF-basic (154 a.a.)	PeproTech	Cat# 100-18B
Animal-Free Recombinant Human EGF	Peprotech	Cat# AF-100-15
Bovine Insulin Powder	Gemini	Cat# 700-112P
Human Plasma Fibronectin Purified Protein	MilliporeSigma	Cat# FC01010MG
Accutase	Sigma-Aldrich	Cat# A6964-100ML
Bovine Serum Albumin (BSA), Fraction V—Serum Replacement Grade	Gemini Bio-Products	Cat# 700-104P
Recombinant Human/Murine/Rat BMP-2 (E.coli derived)	PeproTech	Cat# 120-02
CHIR-99021 (CT99021) HCl	Selleck Chemicals	Cat# S2924
DMEM/High glucose with L-glutamine, sodium pyruvate	Cytiva (formerly GE Healthcare)	Cat# SH30243.01
Corning ITS+ Premix Universal Culture Supplement	Corning	Cat# 354352
Sodium pyruvate	Life Technologies	Cat# 11360070
Ascorbic acid	Sigma-Aldrich	Cat# A4403-100MG
Dexamethasone	Thermo Fisher Scientific	Cat# AAA1759003
Recombinant Human TGF-β3	PeproTech	Cat# 100-36E
Y-27632 RHO/ROCK pathway inhibitor	Stem Cell Technologies	Cat# 72304
KnockOut DMEM	GIBCO	Cat# 10829018
Alcian Blue 8GX	Sigma-Aldrich	Cat# A3157-10G
cOmplete, EDTA-free Protease Inhibitor Cocktail	MilliporeSigma	Cat# 11873580001
Blasticidin (Solution), 100 mg	Invivogen	Cat# NC9016621
QuickExtract DNA Extraction Solution	Lucigen	QE09050

**Bacterial and Virus Strains**		

Adeno-flippase (Ad5CMVFlpO)	Fred Hutchinson Cancer Research Center	VVC-U of Iowa-530 (MTA)

**Critical Commercial Assays**		

NEBNext Ultra II DNA Library Prep Kit for Illumina	New England BioLabs	Cat# E7645S
SeqCap EZ Accessory Kit v2	Roche	Cat# 07145594001
SeqCap EZ Hybridization and Wash Kit	Roche	Cat# 05634261001
SeqCap EZ HE-Oligo Kit A	Roche	Cat# 06777287001
KAPA Library Quantification Kit Illumina platforms, qPCR Master Mix optimized for LightCycler 480	Kapa Biosystems	Cat# KK4854
TRIzol Reagent	Invitrogen	Cat# 15596018
FuGENE 6	Promega	Cat# E2691
NEBNext Multiplex Oligos for Illumina kit	New England BioLabs	Cat# E7335S
AMPure XP	Beckman Coulter	Cat# A63881
Dynabeads mRNA Purification Kit (for mRNA purification from total RNA preps)	Invitrogen	Cat# 61006
Dynabeads Protein G for Immunoprecipitation	Invitrogen	Cat# 10004D
NEBNext Multiplex Oligos for Illumina (Dual Index Primers Set 1)	New England BioLabs	Cat# E7600
Qubit dsDNA HS Assay Kit	Invitrogen	Cat# Q32854
SuperScript IV VILO Master Mix with ezDNase Enzyme	Invitrogen	Cat# 11766050
Dual-Luciferase Reporter Assay System	Promega	Cat# E1960
HiScribe T7 Quick High Yield RNA Synthesis Kit	New England BioLabs	Cat# E2050S
MEGAclear Transcription Clean-up kit	Ambion	Cat# AM1908

**Deposited Data**		

ChIP-seq, ATAC-seq, RNA-seq and Capture-C data	This paper	GEO: GSE145327
H9 hESC 10X Genomics linked-read sequencing	This paper	Sequence Read Archive (SRA) BioProject: PRJNA648128

**Experimental Models: Cell Lines**		

Human: Female H9 human embryonic stem cells (hESCs)	WiCell	WA09; RRID: CVCL_9773

**Experimental Models: Organisms/Strains**		

Mouse: C57BL/6J	The Jackson Laboratory	RRID: IMSR_JAX:000664
Mouse: FVB/NJ	The Jackson Laboratory	RRID: IMSR_JAX:001800
Mouse: B6.Cg-E2f1Tg(Wnt1-cre)2Sor/J	The Jackson Laboratory (Lewis et al., 2013)	RRID: IMSR_JAX:022501
Mouse: B6.129S7-Sox9tm2Crm/J	The Jackson Laboratory (Akiyama et al., 2002)	RRID: IMSR_JAX:013106
Mouse: FVB-mEC1.45del-founder1	This paper	N/A
Mouse: FVB-mEC1.45del-founder2	This paper	N/A

**Oligonucleotides**		

Primers for qRT-PCR, CRISPR-Cas9, LNA probes, see Table S5	This paper	N/A
RNA sequence: mEC1.45 upstream guide RNA1 (E1-45del_sg2U): AACAAGGTAGCGCCTCCTTA	This paper	N/A
RNA sequence: mEC1.45 downstream guide RNA1 (E1-45del_sg2D): ATATCAAGCACAAGGAGTGC	This paper	N/A
RNA sequence: mEC1.45 upstream guide RNA2 (CR50_sg3U): gatgttatggaaccttaagg	This paper	N/A
RNA sequence: mEC1.45 downstream guide RNA2 (CR53_sg3D): gaacaattacaaccaaacag	This paper	N/A

**Recombinant DNA**		

Super piggyBac Transposase expression vector	System Biosciences (SBI)	Cat# PB210PA-1
Plasmid: pGL3-SV40_control	Promega	N/A
Plasmid: pRL	Promega	N/A
Plasmid: pGL3-noSV40-humanEC1.45	This paper	N/A
Plasmid: pGL3-noSV40-humanEC1.45_p300peak1	This paper	N/A
Plasmid: pGL3-noSV40-humanEC1.45_p300peak2	This paper	N/A
Plasmid: pGL3-noSV40-humanEC1.45_p300peak1-2	This paper	N/A
Plasmid: pGL3-noSV40-humanEC1.45_min1	This paper	N/A
Plasmid: pGL3-noSV40-humanEC1.45_min2	This paper	N/A
Plasmid: pGL3-noSV40-humanEC1.45_min1-2	This paper	N/A
Plasmid: pGL3-noSV40-humanEC1.35_S1-2	This paper	N/A
Plasmid: pGL3-noSV40-humanEC1.35_S1	This paper	N/A
Plasmid: pGL3-noSV40-humanEC1.35_S2	This paper	N/A
Plasmid: pGL3-noSV40-humanEC1.25_S3-4-5-6	This paper	N/A
Plasmid: pGL3-noSV40-humanEC1.25_S3	This paper	N/A
Plasmid: pGL3-noSV40-humanEC1.25_S4	This paper	N/A
Plasmid: pGL3-noSV40-humanEC1.25_S5	This paper	N/A
Plasmid: pGL3-noSV40-humanEC1.25_S6	This paper	N/A
Plasmid: pGL3-noSV40-humanCOL2A1enhancer	This paper	N/A
Plasmid: pGL3-noSV40-humanEC1.45_min1-2_4XCoordinatorMutant	This paper	N/A
Plasmid: pGL3-noSV40-humanEC1.45_min1-2_1XCoordinatorMutant	This paper	N/A
Plasmid: pGL3-noSV40-humanEC1.45_min1-2_3XCoordinatorMutant	This paper	N/A
Plasmid: pGL3-noSV40-EC1.45-human_min1-mouse_min2	This paper	N/A
Plasmid: pGL3-noSV40-EC1.45-human_min1-opossum_min2	This paper	N/A
Plasmid: pGL3-noSV40-EC1.45-human_min1-platypus_min2	This paper	N/A
Plasmid: pGL3-noSV40-EC1.45-human_min1-chicken_min2	This paper	N/A
Plasmid: pGL3-noSV40-EC1.45-human_min1-lizard_min2	This paper	N/A
Plasmid: pGL3-noSV40-EC1.45-human_min1-frog_min2	This paper	N/A
Plasmid: pGL3-noSV40-EC1.45-human_min1-coelacanth_min2	This paper	N/A
Plasmid: pGL3-noSV40-mouseEC1.45	This paper	N/A
Plasmid: pGL3-noSV40-humanEC1.45_p300peak1-2_del1	This paper	N/A
Plasmid: pGL3-noSV40-humanEC1.45_p300peak1-2_del2	This paper	N/A
Plasmid: pGL3-noSV40-humanEC1.45_p300peak1-2_del3	This paper	N/A
Plasmid: pGL3-noSV40-humanEC1.45_p300peak1-2_del4	This paper	N/A
Plasmid: pGL3-noSV40-humanEC1.45_p300peak1-2_del5	This paper	N/A
Plasmid: pGL3-noSV40-humanEC1.45_p300peak1-2_del6	This paper	N/A
Plasmid: pGL3-noSV40-humanEC1.45_p300peak1-2_del7	This paper	N/A
Plasmid: pGL3-noSV40-humanEC1.45_p300peak1-2_del8	This paper	N/A
Plasmid: pGL3-noSV40-humanEC1.45_p300peak1-2_del9	This paper	N/A
Plasmid: pGL3-noSV40-humanEC1.45_p300peak1-2_del10	This paper	N/A
Plasmid: pGL3-noSV40-humanEC1.45_p300peak1-2_del11	This paper	N/A
Plasmid: pGL3-noSV40-humanEC1.45_p300peak1-2_del12	This paper	N/A
Plasmid: pGL3-noSV40-humanEC1.45_p300peak1-2_CoordinatorMutant#1	This paper	N/A
Plasmid: pGL3-noSV40-humanEC1.45_p300peak1-2_CoordinatorMutant#2	This paper	N/A
Plasmid: pGL3-noSV40-humanEC1.45_p300peak1-2_CoordinatorMutant#3	This paper	N/A
Plasmid: pGL3-noSV40-humanEC1.45_p300peak1-2_CoordinatorMutant#4	This paper	N/A
Plasmid: pGL3-noSV40-humanEC1.45_p300peak1-2_CoordinatorMutant#5	This paper	N/A
Plasmid: pGL3-noSV40-humanEC1.45_p300peak1-2_CoordinatorMutant#6	This paper	N/A
Plasmid: pGL3-noSV40-humanEC1.45_p300peak1-2_CoordinatorMutant#7	This paper	N/A
Plasmid: pGL3-noSV40-humanEC1.45_p300peak1-2_4XCoordinatorMutant#1-2-3-4	This paper	N/A
Plasmid: pGL3-noSV40-humanEC1.45_p300peak1-2_3XCoordinatorMutant#5-6-7	This paper	N/A
Plasmid: pGL3-noSV40-humanEC1.45_p300peak1-2_7XCoordinatorMutant	This paper	N/A
Plasmid: pHsp68-LacZ-P2A-tdTomato-coreinsulator	This paper	N/A
Plasmid: pHsp68-LacZ-P2A-tdTomato-coreinsulator_humanEC1.45	This paper	N/A
Plasmid: pHsp68-LacZ-P2A-tdTomato-coreinsulator_humanEC1.35-S1-2	This paper	N/A
Plasmid: pHsp68-LacZ-P2A-tdTomato-coreinsulator_humanEC1.25-S3x3	This paper	N/A
Plasmid: pHsp68-LacZ-P2A-tdTomato-coreinsulator_humanEC1.25-S4x3	This paper	N/A
Plasmid: pHsp68-LacZ-P2A-tdTomato-coreinsulator_humanEC1.25-S5x3	This paper	N/A
Plasmid: pHsp68-LacZ-P2A-tdTomato-coreinsulator_humanEC1.25-S6x3	This paper	N/A
Plasmid: pHsp68-LacZ-P2A-tdTomato-coreinsulator_mouseEC1.45	This paper	N/A
Plasmid: pHsp68-LacZ-P2A-tdTomato-coreinsulator_mouseEC1.25-S3x3	This paper	N/A
Plasmid: pHsp68-LacZ-P2A-tdTomato-coreinsulator_mouseEC1.25-S5x3	This paper	N/A
Plasmid: pHsp68-LacZ-P2A-tdTomato-coreinsulator_mouseEC1.25-S6x3	This paper	N/A
Plasmid: Sox9 *in situ* plasmid	From Ian Welsh	N/A
Plasmid: pX458-dual-U6prom-sgRNA-EC1.45_CAGprom-Cas9-GFP	This paper	N/A
Plasmid: EC1.45-HAs_FRT-EF1a-mCherry-T2A-Blast-FRT-FRT3	This paper	N/A
Plasmid: pX458-U6prom-sgRNA-EC1.25_CAGprom-Cas9-GFP	This paper	N/A
Plasmid: EC1.25-firstDonor_HAs-hUbCprom-eGFP-tCD8	This paper	N/A
Plasmid: EC1.25-secondDonor_HAs_only	This paper	N/A

**Software and Algorithms**		

CHOPCHOP	Labun et al., 2019	https://chopchop.cbu.uib.no/
Benchling	Benchling [Biology Software]. (2017)	https://www.benchling.com/
Bruker Recon software	Bruker	N/A
Amira software	ThermoFisher Scientific	https://www.thermofisher.com/us/en/home/industrial/electron-microscopy/electron-microscopy-instruments-workflow-solutions/3d-visualization-analysis-software/amira-life-sciences-biomedical.html
The R package for Statistical Computing	R Core Team (2019); R version 3.6.0	https://www.r-project.org/
R Geomorph package	Adams and Otárola-Castillo, 2013	https://cran.r-project.org/web/packages/geomorph/index.html
R hotelling.test function		https://cran.r-project.org/web/packages/Hotelling/Hotelling.pdf
skewer	Jiang et al., 2014	https://github.com/relipmoc/skewer
bowtie2	Langmead and Salzberg, 2012	http://bowtie-bio.sourceforge.net/bowtie2/index.shtml
bedtools	Quinlan and Hall, 2010	https://github.com/arq5x/bedtools2
bedgraphToBigWig		https://github.com/ENCODE-DCC/kentUtils
macs1.4	Zhang et al., 2008	https://github.com/macs3-project/MACS
cutadapt	Martin, 2011	https://cutadapt.readthedocs.io/en/stable/
HISAT2	Kim et al., 2019	https://daehwankimlab.github.io/hisat2/
featureCounts (subread package)	Liao et al., 2014	http://subread.sourceforge.net/
CapSequm2	Hughes et al., 2014	http://apps.molbiol.ox.ac.uk/CaptureC/cgi-bin/CapSequm.cgi
Long Ranger (longranger-2.2.2)	10X Genomics	https://support.10xgenomics.com/genome-exome/software/pipelines/latest/what-is-long-ranger
Macs2 (macs2 2.1.1.20160309)	Zhang et al., 2008	https://github.com/macs3-project/MACS
SeqPos (Cistrome Project)	Liu et al., 2011	http://cistrome.org/
ggseqlogo	Wagih, 2017	https://cran.r-project.org/web/packages/ggseqlogo/ggseqlogo.pdf
ggplot2	Wickham, 2016	https://ggplot2.tidyverse.org/
UCSC	Kent et al., 2002	https://genome.ucsc.edu/
Samtools (v1.3.1)	Li et al., 2009	http://samtools.sourceforge.net/
Bioanalyzer 2100 Expert Software	Agilent	https://www.agilent.com/en/product/automated-electrophoresis/bioanalyzer-systems/bioanalyzer-software/2100-expert-software-228259
QuantaSoft Software	BioRad	https://www.bio-rad.com/en-us/sku/1864011-quantasoft-software-regulatory-edition?ID=1864011

**Other**		

Leica imaging stereoscope	Leica	N/A
Covaris sonicator E220	Covaris	N/A
Bruker Skyscan 1276 MicroCT (purchased with an NIH S10 Shared Instrumentation Grant, 1S10OD02349701, PI Timothy C. Doyle)	Bruker	https://www.bruker.com/products/microtomography/in-vivo-micro/skyscan-1276/overview.html
Veritas Microplate Luminometer	Turner Biosystems	N/A
Leica M205 FA Stereo Microscope coupled to a Leica DFC7000T digital camera	Leica	N/A
Leica MZ16 microscope coupled to a Leica DFC420 digital camera	Leica	N/A
QX200 Droplet Generator	BioRad	https://www.bio-rad.com/en-gu/sku/1864002-qx200-droplet-generator?ID=1864002
QX200 Droplet Reader	BioRad	https://www.bio-rad.com/en-us/sku/1864003-qx200-droplet-reader?ID=1864003
Chromium controller	10X Genomics	https://www.10xgenomics.com/instruments/chromium-controller/
HiSeq4000 (purchased with funds from NIH under award number S10OD018220)	Illumina	N/A
NextSeq500	Illumina	N/A
Amaxa 4D nucleofector	Lonza	https://bioscience.lonza.com/lonza_bs/US/en/Transfection/p/000000000000203684/4D-Nucleofector-Core-Unit
A&D Weighing EJ-120 Newton Portable Balance, 120 g x 0.01 g; 115V	A&D Weighing	N/A
LightCycler 480	Roche	N/A
High Resolution Episcopic Microscope	Tim Mohun lab	N/A
Bioanalyzer	Agilent	https://www.agilent.com/en/product/automated-electrophoresis/bioanalyzer-systems/bioanalyzer-instrument/2100-bioanalyzer-instrument-228250

### Resource Availability

#### Lead Contact

Further information and requests for resources and reagents should be directed to and will be fulfilled by the Lead Contact, Joanna Wysocka (wysocka@stanford.edu).

#### Materials Availability

DNA constructs and other research reagents generated by the authors will be distributed upon request to other researchers.

#### Data and Code Availability

The accession number for Gene Expression Ombnibus (GEO) where the ChIP-seq, ATAC-seq, and RNA-seq datasets generated in this study are available is: GSE145327 (https://www.ncbi.nlm.nih.gov/geo/query/acc.cgi?acc=GSE145327). The accession number for the Sequence Read Archive (SRA) BioProject where the 10X Genomics linked-read sequencing data is available is: PRJNA648128.

### Experimental Model and Subject Details

#### Mouse models and husbandry

C57BL/6J (RRID: IMSR_JAX:000664), FVB/NJ (RRID: IMSR_JAX:001800), C57BL/6J *Wnt1::Cre2* (RRID: IMSR_JAX:022501) (Lewis et al., 2013) and C57BL/6J *Sox9* flox (RRID: IMSR_JAX:013106) (Akiyama et al., 2002) mice were obtained from Jackson Labs and mEC1.45 deletion lines were generated and characterized on the FVB background as described in Method Details. Mice were housed in RAFII facility at Stanford University, with free access to food and water. All animal protocols were approved by the Administrative Panel on Laboratory Animal Care at Stanford University. Of note, the second generation *Wnt1::Cre2* driver avoids ectopic Wnt activation and impact on midbrain development, which might have confounded studies performed with the first-generation *Wnt1::Cre* driver (Lewis et al., 2013). Only females were used to propagate the *Wnt1::Cre2* driver.

For breeding, two female mice were introduced into a cage with a single male and monitored for timed pregnancies. To generate compound heterozygous mice carrying a deletion of mEC1.45 on one allele and a conditionally deletable *Sox9* gene on the other allele, we crossed the mEC1.45 enhancer deletion lines to *Wnt1::Cre2* driver females, and then crossed the resultant *Wnt1::Cre2;mEC1.45del/+* females to *Sox9F/F* males. Mice were genotyped by tail clipping, lysis with Proteinase K in tail buffer (0.2M NaCl, 0.2% SDS, 0.05M EDTA and 0.1M Tris-HCl pH 8.0), precipitation with isopropanol, and analytical PCR using genotype-specific primers and Dream Taq Master Mix (Thermo Fisher Scientific). To monitor weight-gain, mice were weighed from birth to post-natal day 25 (P0-P25) using a scale with 2 decimal places (accuracy ± 0.01 g). Males and females were included for embryonic assays at E9.5 and E11.5, and for post-natal weighing.

#### Culture of H9 human embryonic stem cells (hESCs)

Female H9 (WA09; RRID: CVCL_9773) human embryonic stem cells (hESCs) were obtained from ATCC and cultured in mTeSR (Stem Cell Technologies) and grown on Matrigel Growth Factor Reduced (GFR) Basement Membrane Matrix (Corning) at 37°C. hESCs were fed every day and passaged every 5-6 days using ReLeSR (Stem Cell Technologies).

### Method Details

#### Mouse genome editing using CRISPR/Cas9

Mouse orthologous mEC1.45 was deleted *in vivo* using CRISPR-Cas9 editing in the FVB strain as previously described (Osterwalder et al., 2018). Briefly, pairs of sgRNAs were designed to target upstream and downstream of the enhancer sequence to be deleted using CHOPCHOP (Labun et al., 2016) and Benchling (https://www.benchling.com/). sgRNAs were generated using a modified version of a previously published oligo assembly protocol (Varshney et al., 2015). In this process an oligo encoding a T7 promoter and the guide RNA sequence were annealed to a second, generic oligo, and Phusion polymerase (NEB) was used for extension. The guide RNA was synthesized using HiScribe T7 Quick High Yield RNA Synthesis kit (NEB) and purified using the MEGAclear Transcription Clean-up kit (Ambion) prior to quantification. A mix containing Cas9 protein (final concentration of 20 ng/ul; IDT Cat. No. 1074181) and four sgRNAs (12.5 ng/μL each) in an injection buffer (10 mM Tris, pH 7.5; 0.1 mM EDTA) was injected into the pronucleus of FVB mouse embryos at the single-cell stage. F0 founder mice were genotyped using primers spanning the desired deletion region and High-Fidelity Platinum Taq polymerase (Thermo Fisher Scientific) to identify deletion breakpoints, which were validated and mapped using Sanger sequencing. Deletions were validated in second generation F1 animals, and heterozygous animals were crossed to generate homozygous and heterozygous animals for breeding. Two founder lines were established with deletions differing by 58bp. Founder 1 has a 3572bp deletion, and Founder 2 has a 3630bp deletion.

#### Generation of CRISPR/Cas9 genome-edited cell lines

Human ESCs were targeted for enhancer deletion using two strategies. In the first strategy, H9 hESCs were transfected using FuGENE 6 (Promega) with a targeting construct containing Blasticidin selection cassette, flanked by FRT sites, and homology arms for either side of EC1.45 along with a plasmid encoding Cas9 plus single guide RNAs (sgRNAs) flanking EC1.45. Transfected hESCs were grown to confluency and split onto a new plate before selection with 1 μg/mL Blasticidin until all cells died on a mock/GFP transfected control well. Surviving colonies were picked into a 48-well plate, expanded, split and screened for enhancer deletion using a genomic primer and a primer in the targeting cassette. Heterozygous enhancer deleted clones were infected with 1E+08 pfu/mL Adeno-flippase (Ad5CMVFlpO, Fred Hutchinson Cancer Research Center) and clones screened for excision of the selection cassette by PCR. For screening, genomic DNA was extracted using QE buffer (Lucigen) and PCR was performed using Q5 polymerase (NEB). Heterozygous enhancer deletions were generated to be in keeping with the heterozygous deletions seen in PRS patients, and to enable allele-specific *SOX9* gene expression analysis.

In the second targeting strategy, a scar-less editing methodology was performed (Ikeda et al., 2018). A targeting construct was designed to insert adjacent to EC1.25 in H9 hESCs, containing a hUbC promoter driving expression of an eGFP-T2A-tCD8 cassette. H9 hESCs were nucleofected with 3 μg this construct and 3 μg of a plasmid encoding Cas9 and a single guide RNA targeting one side of EC1.25 using an Amaxa 4D nucleofector (pulse code CB150). GFP positive cells were isolated by FACS after 7-12 days and plated onto a 6-well plate. GFP positive colonies were then picked, expanded and screened for integration of the targeting cassette using primers within the targeting cassette and flanking genomic sequence. Genomic DNA was extracted using QE buffer (Lucigen) and PCR screening was performed using PrimeSTAR GXL DNA Polymerase (Takara) and heterozygous targeted clones were isolated. A second cassette was designed to excise the first targeting construct as well as EC1.25 using enhancer-flanking homology arms with no extra exogenous sequences, to leave a scar-less deleted enhancer region. To generate matched wild-type clones, a wild-type homology template was used to excise the targeting cassette. Colonies were selected by screening for loss of tCd8 by magnetic activated sorting (MACS), plated onto a 6-well plate, and following dilute re-plating, colonies were picked into a 48-well plate. Clones were passaged and screened using primers flanking EC1.25 to identify positive clones with EC1.25 deleted on one allele, or excision of the targeting construct for the matched wild-type controls. Genomic DNA was extracted using QE buffer (Lucigen) and PCR screening was performed using Q5 polymerase (NEB).

#### Differentiation of hESC to hCNCCs and chondrocytes

hESCs were differentiated to human cranial neural crest cells (hCNCCs) using a protocol described previously (Prescott et al., 2015). Briefly, hESCs were grown for 5-6 days until large colonies formed, then were disaggregated using collagenase IV and gentle pipetting. Clumps of ~200 hESCs were washed in PBS and transferred to a 10cm Petri dish in neural crest differentiation media (NDM). NDM: 1:1 ratio of DMEM-F12 and Neurobasal, 0.5x Gem21 NeuroPlex Supplement With Vitamin A (Gemini, 400-160), 0.5x N2 NeuroPlex Supplement (Gemini, 400-163), 1x antibiotic/antimycotic, 0.5x Glutamax, 20ng/ml bFGF (PeproTech, 100-18B), 20ng/ml EGF (PeproTech, AF-100-15) and 5ug/ml bovine insulin (Gemini Bio-Products, 700-112P). After 7-8 days, neural crest emerged from neural spheres attached to the Petri dish, and after 11 days, neural crest cells were passaged onto fibronectin-coated 6-well plates using accutase and fed with neural crest maintenance media (NMM). NMM: 1:1 ratio of DMEM-F12 and neurobasal, 0.5x Gem21 NeuroPlex Supplement with Vitamin A (Gemini, 400-160), 0.5x N2 NeuroPlex Supplement (Gemini, 400-163), 1x antibiotic/antimycotic, 0.5x Glutamax, 20ng/ml bFGF, 20ng/ml bFGF EGF and 1mg/ml BSA (Gemini). After 2-3 days, neural crest cells were split 1:3 and the following day cells were fed with neural crest long-term media. Long term media: neural crest maintenance media + 50pg/ml BMP2 (PeproTech, 120-02) + 3uM CHIR-99021 (Selleck Chemicals, S2924) (BCh media). hCNCCs were then passaged twice to passage 4 when the majority of assays were performed, or cells were further differentiated to chondrocytes.

To differentiate hCNCCs to chondrocytes, passage 3 hCNCCs were passaged to passage 4, and the following day were transitioned to chondrocyte media without TGFb3 (ChM). ChM: DMEM-HG, 5% FBS, 1x ITS premix, 1mM sodium pyruvate, 50 μg/mL ascorbic acid, 0.1 μM dexamethasone and 1x antibiotic/antimycotic. The following day, cells were fed with chondrocyte media with TGFb3 (ChMT). ChMT: ChM + 10 ng/mL TGFb3. Cells were fed every subsequent 3 days with ChMT. Cells were harvested at day 5 and/or 9 of the differentiation for the majority of assays.

To evaluate the chondrogenic differentiation, we performed qRT-PCR for two independent experiments, and assessed the differentiation from P4 hCNCCs to Day 9 of chondrogenic differentiation. *COL2A1* and Aggrecan (*ACAN*) are known to be directly regulated by SOX9 during chondrogenesis, *COL2A1* is an early marker of the chondrocyte lineage, while *ACAN* is a marker of overtly differentiated chondrocytes. *SOX5* and *SOX6* are two SOX family transcription factors that are co-expressed with *SOX9* in chondrocytes, and all three factors often form a trio at regulatory elements to promote chondrocyte differentiation. Notably, *SOX5* is induced early during our *in vitro* differentiation. *BMP2* is a marker of hypertrophic chondrocytes and is essential for chondrocyte proliferation and maturation.

#### Alcian Blue Staining

Alcian Blue stains extracellular matrix proteoglycan components associated with chondrocytes. hCNCCs were differentiated to chondrocytes and fixed with 4% PFA for 15 minutes. Cells were washed three times with 1x PBS, and incubated overnight in 20% sucrose at 4°C. The following day, cells were stained with Alcian Blue solution (pH 2.5) for 30 minutes. Alcian Blue solution was prepared by diluting 1g Alcian Blue, 8GX in 100mL 3% Acetic Acid solution, pH was adjusted to 2.5 with acetic acid. Following Alcian Blue staining, cells were washed with 3% acetic acid for 3 minutes, followed by several washes with 95% ethanol. Ethanol was removed and cells were imaged.

#### Capture-C

Capture-C was performed as previously described (Davies et al., 2016). Briefly, cells were crosslinked for 10 min in 2% formaldehyde in PBS, quenched with 125mM glycine for 5 min, scraped, collected and pelleted by centrifugation (500 r*cf.*, 5 min, 4°C). Cells were washed with 5mL cold PBS, pelleted and resuspended in 5mL cold lysis buffer (10mM Tris pH8, 10mM NaCl, 0.2% Ipegal CA-630 in water with cOmplete Protease Inhibitor Cocktail) for 20 min. Cells were pelleted, washed with 5mL cold PBS, pelleted, resuspended in 1mL cold PBS, flash frozen in liquid nitrogen and stored at −80°C. For 3C library preparation, samples were defrosted on ice, pelleted and resuspended in 1xDpnII digestion buffer before extended digestion with DpnII overnight with addition of extra enzyme. DpnII was then heat inactivated at 65°C and samples were ligated with T4 Ligase for ~22 hours. DNA was extracted by sequential Proteinase K and RNase digestion followed by phenol-chloroform isoamylalcohol extraction and precipitation with ethanol and sodium acetate. Efficiency of digestion and ligation was assessed by gel electrophoresis and digestion efficiency was further assessed by qPCR. For addition of Illumina sequencing adaptors, samples were sheared by Covaris sonication and purified by XP SPRI bead clean-up. Sequencing adaptors were annealed using NEB Ultra II kit. Libraries were PCR amplified using Herculase II polymerase (Agilent) in duplicate to add indexing primers and purified by XP SPRI bead clean-up. Samples were pooled and quantified using Qubit dsDNA BR assay kit.

Biotinylated oligos for capture were designed using the Capsequm online tool (http://apps.molbiol.ox.ac.uk/CaptureC/cgi-bin/CapSequm.cgi) and ordered from IDT. 1-2 μg indexed 3C library was mixed with 5 μg COT DNA, 1nmol TS-HE Universal Oligo and 1nmol of TS-HE Index Oligo (Nimblegen SeqCap EZ HE-oligo and Accessory kit) and dried by vacuum centrifugation at 55°C until completely dry. The 3C library plus blocking oligos were then carefully resuspended in 7.5 μL 2X hybridization buffer and 3 μL Hybridization buffer A and denatured at 95°C for 10min. The 3C library was then transferred into a preheated PCR tube at 47°C containing 4.5 μL of pooled Biotinylated oligonucleotide capture probes at 2.9 μM. After a brief mix and centrifugation, the 3C library oligo mix was incubated at 47°C for 18-20 hr. Following this incubation, the 3C library was washed and recovered by streptavidin bead (M-270 Dynabeads, Invitrogen) pull down using the Nimblegen SeqCap EZ Hybridization and wash kit. Following the recovery of the captured material, the captured DNA was amplified on the streptavidin beads using KAPA HiFi HotStart ReadyMix and POST-LM PCR oligo 1&2 and purified by XP SPRI bead clean-up. For improved capture efficiency, a second round of capture was performed on the total amplified DNA from the first capture. Following the second round of capture, the library was quantified using KAPA library quantification kit using the average size calculated from Bioanalyzer. Libraries were then sequenced on Illumina HiSeq-4000 (2x 150bp).

#### RNA isolation and preparation of RNA-seq libraries

Total RNA was extracted from a 6-well of hESC, early (P1 and 2) and late (P3 and P4) hCNCCs and day 9 chondrocytes differentiated from hCNCCs using Trizol reagent (Invitrogen) for four independent differentiations. 10 μg RNA was purified twice by Dyna1 oligo(dT) beads (Invitrogen) to enrich for poly(A)^+^ mRNA. The mRNA was then fragmented using 10X fragmentation buffer (Ambion) for exactly 5 min and purified by ethanol precipitation with sodium acetate and RNase-free glycogen. First strand synthesis was performed using Random Hexamer Primers (Invitrogen) and SuperScript II (Invitrogen), followed by second strand synthesis using DNA PolI and RNaseH (Invitrogen), and cDNA was purified using Nucleospin Gel and PCR Cleanup (Takara). All of the cDNA was used for library preparation by end repair, A-tailing and adaptor ligation (NEB). The samples were treated with USER enzyme, purified using XP SPRI beads then subjected to dual size selection using XP SPRI beads using bead ratios 0.55x to remove > 700bp followed by 0.85x to recover > 200bp sized cDNA. Size-selected cDNA was amplified using NEBNext HiFi 2X PCR mix and Dual Index Primers (NEB, E7600) for 7-10 cycles (as determined by qPCR). Libraries were then purified using XP SPRI beads, and quantified using Qubit dsDNA HS assay kit and pooled for sequencing using average library size (bp) from Bioanalyzer and concentration from KAPA quantification (Kapa Biosystems). Libraries were sequenced using NextSeq 500 (2x 75 bp).

#### 10X Genomics Linked-Read sequencing

High molecular weight genomic DNA (HMW gDNA) was generated from H9 hESCs by the salting out method (10x Genomics, manual CG000116) and quality was checked on a FEMTO pulse instrument (Agilent). Linked read libraries were prepared according to the manufacturer’s instructions (10x Genomics, manual CG00043) and sequenced on a HiSeq 4000 instrument (2 lanes, 2x 150 bp).

#### cDNA preparation and reverse transcriptase digital droplet PCR (RT-ddPCR)

Total RNA was extracted from early (Day 11, P1 and 2) and late (P3 and P4) hCNCCs and day 5 and 9 chondrocytes differentiated from hCNCCs for wild-type or enhancer mutant cell-lines using Trizol reagent (Invitrogen) for at least four differentiations, or from dissected craniofacial prominences from at least four embryos. 100ng – 1ug RNA was used to generate cDNA using the SuperScript Vilo IV MasterMix with ezDNase enzyme (Invitrogen). Primers and locked nucleic acid (LNA) probes were designed by IDT’s custom design service to the human *SOX9* or mouse *Sox9* 3′UTR. For H9 hESC samples, LNA probes were centered on the rs74999341 T/C SNP – a HEX LNA probe detects the T-allele and FAM detects the C-allele. For mouse samples, LNA probes were centered on a G/C SNP in the C57BL/6J versus FVB mouse strains respectively – a HEX LNA probe detects the G-allele and FAM detects the C-allele. cDNA dilution factor was determined using qPCR with 1X PrimeTime Gene Expression Master Mix (IDT), 500nM primers and 250nM probes, run on LightCycler 480 (Roche). ddPCR reactions were performed using diluted cDNA (10-100X diluted), 900nM primers and 250nM probes and 1X ddPCR Supermix for probes (no dUTP, BioRad). ddPCR droplets were generated using the QX200 Droplet Generator (BioRad) and droplets were read using QX200 Droplet Reader (BioRad) and analyzed using the QuantaSoft Software (BioRad).

#### Chromatin immunoprecipitation (ChIP)

5-15 million cells were cross-linked per ChIP experiment in 2mL PBS per 6-well with 1% methanol-free formaldehyde for 5-10 min and quenched with a final concentration of 0.125M glycine for 5 min with nutation. Cross-linked cells were washed with PBS, scraped and pelleted by centrifugation, flash-frozen in liquid nitrogen and stored at −80°C. Samples were defrosted on ice and resuspended in 5mL LB1 (50 mM HEPES-KOH pH 7.5, 140 mM NaCl, 1 mM EDTA, 10% glycerol, 0.5% NP-40, 0.25% Triton X-100, with 1X cOmplete Protease Inhibitor Cocktail and optionally 1mM PMSF) and rotated vertically for 10 min at 4°C. Samples were centrifuged for 5 min at 1350 x g at 4°C, and resuspended in 5mL LB2 (10 mM Tris, 200 mM NaCl, 1 mM EDTA, 0.5 mM EGTA, with 1X cOmplete Protease Inhibitor Cocktail and optionally 1mM PMSF) and rotated vertically for 10 min at 4°C. Samples were centrifuged for 5 min at 1350 x g at 4°C, and resuspended in 1mL LB3 per 10 million cells (maximum concentration of cells for Covaris sonication), or 1 mL per ChIP. Samples were sonicated in 1mL AFA tubes for 5 min on E220 evolution Covaris with settings Peak power = 140, Duty Factor = 10, Cycles per burst = 200 to achieve chromatin sized approximately 500-2000bp.

Following sonication, samples were re-combined (if aliquoted for sonication), Triton X-100 was added to the fragmented chromatin to a final concentration of 1%, and the chromatin divided for input (1%–2%) and ChIP samples. 5 μg anti-histone antibody or 5-9 μg anti-transcription factor antibody was added per ChIP sample, and incubated overnight at 4°C. Antibodies used include TWIST1 (Abcam, ab50887), RAD21 (Abcam, ab992), CTCF (Cell Signaling, 2899S), H3K4me1 (Active Motif, 39297), H3K4me3 (Active Motif, 39159), H3K27ac (Active Motif, 39133) and p300 (Santa Cruz). Protein G Dynabeads (ThermoFisher) were first blocked with Block solution (0.5% BSA (w/v) in 1X PBS) and then added to cleared chromatin to bind antibody-bound chromatin for a 4-6 hour incubation. Chromatin-bound Dynabeads were washed at least 6 times with chilled RIPA wash buffer (50 mM HEPES-KOH pH 7.5, 500 mM LiCl, 1 mM EDTA, 1% NP-40, 0.7% Na-Deoxycholate), followed by a wash with chilled TE + 50 mM NaCl. Chromatin was eluted for 15-30 min in Elution Buffer (50 mM Tris, 10 mM EDTA, 1% SDS) at 65°C with frequent vortexing. The ChIP and input samples were then incubated at 65°C overnight to reverse cross-links (12-16 hours). Samples were diluted and sequentially digested with RNase A (0.2 mg/mL) for 2 hours at 37°C followed by Proteinase K (0.2 mg/mL) for 2 hours at 55°C for 2-4 hours to digest protein. ChIP and input samples were purified by phenol-chloroform-isoamylalcohol extraction and precipitation with final concentration 70% ethanol, 0.3M NaOAc pH 5.2 and 1.5 μL glycogen.

For library preparation, samples were quantified by Qubit dsDNA HS assay kit, and 10-30ng of ChIP DNA was used for library preparation with end repair, A-tailing, and adaptor ligation (NEB). Following USER enzyme treatment, samples were purified using Nucleospin Gel and PCR Cleanup (Takara) and separated by gel electrophoresis and size-selected for 220-500 bp by gel extraction. Libraries were then amplified to add indices using NEBNext HiFi 2X PCR mix and NEBNext Multiplex Oligos for Illumina kit (NEB, E7335S) with 9-15 cycles (as determined by qPCR). ChIP libraries were purified by two rounds of XP SPRI bead clean-up to deplete adaptors. Library concentration and quality was assessed by Bioanalyzer (to determine size) and KAPA qPCR was used to pool multiple libraries. Samples were sequenced using NextSeq or HiSeq 4000 platform (2x 75bp).

#### Episomal ChIP-ddPCR

Around 5 million passage 4 hCNCCs were transfected with 1.5 μg wild-type (WT) and 1.5 μg Coordinator mutant (4x mut) luciferase min1-min2 reporter plasmid in 300 μL optimum with 9 μL Fugene-6. Cells were fixed after 24 hours and ChIP performed as described above. 9 μg TWIST antibody was used per ChIP (Abcam, ab50887). Primers were designed to amplify across the min2-plasmid backbone junction, and plasmid-specific probes were designed to distinguish between the wild-type and Coordinator mutant sequences. ChIP and input dilution factors were determined using qPCR with 1X PrimeTime Gene Expression Master Mix (IDT), 500nM primers and 250nM probes, run on LightCycler 480 (Roche). ddPCR reactions were performed using ChIP DNA (40X diluted) and input DNA (640X diluted), 900nM primers and 250nM probes and 1X ddPCR Supermix for probes (no dUTP, BioRad). ddPCR droplets were generated using the QX200 Droplet Generator (BioRad) and droplets read using QX200 Droplet Reader (BioRad) and analyzed using the QuantaSoft Software (BioRad).

#### ATAC-seq

ATAC-seq was performed as described previously (Buenrostro et al., 2013; Corces et al., 2017) Briefly, cells were dissociated and treated with DNaseI (Worthington) and 50,000 viable cells were sorted as DAPI negative. Cells were pelleted at 500 RCF for 5 min at 4°C and resuspended in ATAC-resuspension buffer containing 0.1% NP40, 0.1% Tween20, and 0.01% Digitonin and incubated on ice for 3 minutes. Following wash-out with cold ATAC-Resuspension Buffer (RSB, 10 mM Tris-HCl pH 7.4, 10mM NaCl, 3mM MgCl2 in sterile water) containing 0.1% Tween20, cells were pelleted and resuspended in 50 μL transposition mix (25 μL 2x TD buffer, 2.5 μL transposase (100nM final), 16.5 μL PBS, 0.5 μL 1% digitonin, 0.5 μL 10% Tween20, 5 μL H_2_O) and incubated for 30 minutes at 37°C with shaking. The reaction was purified using the Zymo DNA Clean and Concentrator Kit, and amplified using PCR primers defined in Buenrostro et al. (2013). Libraries were purified using the Zymo DNA Clean and Concentrator Kit, quantified using the KAPA Library Quantification kit and quality assessed by Bioanalyzer (also used to determine average size for pooling libraries). Samples were sequenced on the HiSeq4000 platform (2x 75bp).

#### Cloning PRS locus enhancers for luciferase assay, including Coordinator mutant and vertebrate species min2 sequences

EC1.45, as well as combined constituent enhancers for EC1.35 (S1 and S2) and EC1.25 (S3, S4, S5 and S6) were PCR-amplified from H9 hESC genomic DNA and cloned into the pGL3 luciferase reporter vector. To generate Coordinator mutant sequences, Coordinator motifs were identified in EC1.45 using fimo from the meme suite (Grant et al., 2011). Coordinator motifs were then mutated *in silico* at positions with greatest information content in the PWM, to resemble sequence changes associated with reduction in enhancer activity during human-chimpanzee divergence (Prescott et al., 2015). To synthesize orthologous min2 sequences, Multiz Alignments of 100 Vertebrates (UCSC) was used to identify orthologous sequences for mouse, opossum, platypus, lizard, chicken, frog and coelacanth which were then extended to each be 267 bp long. Sequences containing mutant EC1.45 Coordinator motifs and vertebrate min2 sequences were ordered from TWIST Bioscience (or IDT for coelacanth sequence) and cloned into the pGL3 luciferase reporter vector.

#### Luciferase assay

Luciferase assays were performed as described previously (Prescott et al., 2015). Briefly, H9 hESC were differentiated following the hCNCC differentiation protocol and passaged to passage 3. For hCNCCs, cells were transfected immediately following passaging to passage 4 in 24-well plates. For hESCs, cells were split the day before into a 24 well plate in ROCKi (Y27632) and transfected the following day. For chondrocytes, passage 3 hCNCCs were split into a 24-well plate, and the now passage 4 hCNCCs were transitioned to ChM media for 1 day, and ChMT media for 4 more days before being transfected for the luciferase assay. Transfections were performed in technical triplicate in a single experiment, with each well receiving 10ng of pGL3 plasmid, 0.5ng of control pRL firefly renilla plasmid, 89.5 μL carrier DNA (circularized pGEMT plasmid) and 0.3 μL Fugene 6 in 50 μL of optimum. The pGL3 plasmid contains the firefly luciferase gene driven by an SV40 promoter with either a control SV40 enhancer downstream, or a test enhancer sequence cloned upstream (Promega), the pRL plasmid acts as a transfection control with Renilla luciferase driven by an upstream CMV enhancer and CMV promoter (Promega). 24 hours after transfection, cells were washed in PBS, and lysed in 150 μL 1X passive lysis buffer (in PBS) for 15 min (Promega). 20 μL lysate was then transferred to an opaque flat-bottomed plate for reading with a luminometer (Veritas). An automated injector added 100 μL LARII reagent and the well was read using the following parameters: 2 s delay, 10 s integration. 100 μL Stop-and-Glow reagent was then injected into the well and read using the same parameters. Luciferase assays were repeated in biological duplicate or triplicate with at least two different DNA preps; empty vector and SV40 enhancers were included in each experiment as negative and positive controls, respectively.

#### LacZ *in vivo* reporter assay

A second generation LacZ reporter vector was cloned with a Hsp68 promoter driving expression of LacZ-P2A-tdTomato flanked by core insulator sequences to minimize position effects of site of integration and with a multiple cloning site upstream for cloning enhancer sequences to be tested. EC1.45 was subcloned into this vector from the luciferase reporter, as was S1-S2 combined EC1.35 enhancer cluster. The constituent enhancers from EC1.25 (S3, S4, S5 and S6) were triplexed and inserted into the multiple cloning site. The reporter plasmids were linearized and injected into fertilized mouse oocytes, implanted into recipient females and allowed to develop to two distinct mouse embryonic stages, E9.5 or E11.5, that represent distinct periods of craniofacial development (Cyagen – for EC1.45, EC1.35 and EC1.25-S5; LBNL for remaining constructs). Embryos were harvested and processed for X-gal staining to reveal spatial expression pattern of the LacZ gene under the control of the putative cloned enhancer sequence. To be considered reproducible, craniofacial expression patterns had to be observed in at least three embryos (Visel et al., 2007). Embryos were imaged using a Leica M205 FA Stereo Microscope coupled to a Leica DFC7000T digital camera or a Leica MZ16 microscope coupled to a Leica DFC420 digital camera.

#### Preparation of *in situ* hybridization probes

Murine cDNA probes for *Sox9* corresponding to nucleotides 2443-3588 of RefSeq NM_011448 were TA-cloned from E14.5 C57BL/6 cDNA. The *Sox9* probe was linearized and used to transcribe a DIG-labeled antisense probe using an *in vitro* transcription kit (Roche), following the manufacturer’s instructions.

#### *In situ* hybridization

*In situ* hybridization was performed as described previously (Welsh et al., 2018). Embryos (C57BL/6J) were collected at E9.5 and E11.5, dissected into cold PBS and fixed overnight in 4% PFA in PBS at 4°C. Embryos were dehydrated through a methanol-PBST (PBS + 0.1% Tween20) series and stored at −20°C in 100% methanol. For hybridization, embryos were bleached in 6% hydrogen peroxide in methanol for 1 hour, followed by rehydration, treatment with Proteinase K (10 μg/mL) for 10 minutes at 37°C, and then washed twice in ice cold PBST. Embryos were re-fixed in 4% PFA/0.2% glutaraldehyde in PBST for 20 minutes then washed twice in PBST. Embryos were then transferred to a prehybridization solution for 1 hour at 68°C followed by overnight incubation with the *Sox9* riboprobe at 68°C. The following day, embryos were first rinsed with hybridization buffer, then washed 7 times (30 minutes each) with 2XSSC/50%formamide/0.1% Tween at 68°C. Subsequently, embryos were washed twice with TBST, twice with MABT, and blocked in 10% blocking solution (Roche) diluted in MABT for 1 hour at room temperature. Embryos were then incubated with anti-digoxigenin-AP antibody (Roche) at a concentration of 1:4000 in 1% blocking solution overnight at 4°C with rocking. The following day, embryos were washed extensively in TBST buffer at room temperature for 4 days. Colorimetric detection was performed with BM-Purple Chromogenic Reagent (Roche). Lastly, embryos were washed in PBST and post-fixed with 4% PFA and stored in PBS containing 0.05% sodium azide. Embryos were imaged using a Leica M205 FA Stereo Microscope and a Leica DFC7000T digital camera. A minimum of three embryos were processed per stage.

#### High resolution episcopic microscopy (HREM)

HREM was performed for a representative LacZ reporter embryo for human EC1.45. In HREM, the embedding medium is made highly fluorescent via addition of eosin. The embryo signal is detected as suppression of this fluorescence, in addition chromogenic substrates, for example those used in our LacZ reporter embryos are detected at distinct wavelengths from tissue morphology (Mohun and Weninger, 2012a). The embryo was embedded in JB-4 embedding solution as previously described (Mohun and Weninger, 2012b), sectioned with an SM2500 motorized microtome (Leica) and the block face was concurrently imaged using custom imaging apparatus. Visualization of HREM images was performed in Amira.

#### MicroCT and mandibular morphometry

Mouse embryos were collected at E18.5 of development and fixed in 4% PFA. MicroCT was performed using a Bruker Skyscan at 15um resolution, 0.25mm Al filter, 415ms exposure, 2k resolution and images were acquired every 0.5 degrees for 180 degrees.

### Quantification and Statistical Analysis

#### Analyzing and plotting luciferase data

Multiple independent luciferase experiments were performed with independent plasmid preparations and distinct cell passages or differentiations. The number of independent replicates for each luciferase assay is indicated in the figure legend, or in the plot as n =. For each biological replicate experiments, technical triplicate transfections were performed for each plasmid. Empty vector and SV40 enhancer were included in all experiments as negative and positive controls, respectively. When testing enhancer activity in chondrocytes, an additional *COL2A1* intron1 enhancer was included which is active in both hCNCCs and chondrocytes. To plot multiple luciferase experiments on the same plot, linear regression was performed in R and residuals were plotted as boxplots. For ease of visualization, the median value for the empty vector control was set to 0. Significance of activity change were determined by t-test.

Fimo analysis was used to detect Coordinator motifs in the vertebrate min2 sequences, and the fimo scores were summed to estimate the relative number and match to consensus of Coordinator motifs for each species. The summed fimo score was compared to the luciferase signal and linear regression analysis performed to define the relationship between the two measures and ANOVA was used to determine the significance.

#### Analyzing and plotting ddPCR data

Concentration of the two alleles of *SOX9* or *Sox9* was determined by RT-ddPCR using allele-specific HEX/FAM probes from the QuantaSoft Software. To plot allelic skew, concentration for the mutated allele was divided by the concentration for the wild-type allele and plotted as a ratio (red boxplots). For matched wild-type cells (green boxplots), the same ratio was plotted; this revealed the presence of an allelic skew even in unedited cells suggesting that polymorphisms between the two alleles can drive differences in allelic expression. For Figure 3C, samples were collected for two wild-type cell lines and two mutant EC1.45del/+ cell lines for two independent differentiations. For Figure S4F, samples were collected for five wild-type cell lines and five mutant EC1.25del/+ cell lines for three independent differentiations.

To calculate the % change in *Sox9* expression upon mEC1.45 deletion, the mean allelic skew for the edited embryo facial tissues (mEC1.45del/+) was divided by the mean allelic skew from matched wild-type tissues to account for the normal level of allelic skew detected in unedited wild-type samples. For Figure 6E, four wild-type and four mEC1.45del/+ embryos were dissected at E11.5. For Figure S7H, six wild-type and seven mEC1.45del/+ embryos were dissected at E9.5 (from two litters). Significant differences in allelic skew were determined by t-test.

For TWIST1 ChIP-ddPCR, the concentration of wild-type or Coordinator mutant plasmid were calculated for ChIP and input samples from the QuantaSoft Software using plasmid-specific HEX/FAM probes and primers spanning the min2 sequence and the plasmid backbone. A ratio was calculated between the TWIST ChIP and input samples, and normalized to 1 for the wild-type plasmid, revealing that TWIST1 binding was dramatically reduced on the Coordinator mutant plasmid.

#### External datasets

External next generation sequencing data were downloaded from the Sequence Read Archive

(SRA) and analyzed as below.

#### ChIP-seq analysis

ChIP sequencing reads were trimmed using skewer and aligned to the human genome (hg19) using bowtie2. For normalization, bedgraph files were generated from aligned bam files using bedtools genomecov with -scale option to normalize to 1 million reads. For visualization, bigwig files were generated using bedgraphToBigWig. Peak calling was performed using macs1.4, for replicate experiments the intersect tool from bedtools was used to identify peaks present in both replicates.

#### ATAC-seq analysis

Nextera adaptor sequences were trimmed from ATAC sequencing reads using cutadapt and aligned to the human genome (hg19) using bowtie2. For normalization, bedgraph files were generated from aligned bam files using bedtools genomecov with -scale option to normalize to 1 million reads. For visualization, bigwig files were generated using bedgraphToBigWig. Peak calling was performed using macs1.4, for replicate experiments the intersect tool from bedtools was used to identify peaks present in both replicates.

#### RNA-seq analysis

RNA sequencing reads were trimmed using cutadapt and aligned to human gene models (hg38) using HISAT2. Read counts per gene were quantified using featureCounts, gene expression was normalized to fragments per million and plotted in R.

#### Capture-C analysis

Capture-C analysis was performed using bespoke analysis scripts outlined here: http://userweb.molbiol.ox.ac.uk/public/telenius/captureManual/oligofile.html. For comparison across samples, Capture-C profiles were plotted as the number of unique interactions per restriction fragment normalized to 10,000 interactions in *cis*. For quantification, normalized interactions in *cis* were extracted for DpnII fragments overlapping the feature of interest and plotted as a boxplot in R. Statistical significance was determined by t-test for successive stages of hCNCC differentiation.

#### 10X Linked-Read analysis

10X Linked-Read sequencing data was analyzed using Long Ranger (longranger-2.2.2) and visualized using the 10X loupe genome browser.

#### Motif discovery

To identify *de novo* DNA sequence motifs enriched at TWIST1 binding sites, we called peaks from the TWIST1 ChIP-seq data using MACS2 and identified *de novo* motifs underlying these peaks using the SeqPos tool in Cistrome. Known motifs were identified using the TOM-TOM motif comparison tool (Gupta et al., 2007). Consensus DNA binding motifs were plotted using R package ggseqlogo and plotted using ggplot2.

#### MicroCT mandibular morphometry, quantification and plotting

Reconstruction of microCT data was performed using Bruker Recon software. Hemimandible, pre-maxilla, maxilla, palatine and occipital bones were segmented and landmarks were placed using Amira software (Ho et al., 2015; Welsh et al., 2018). x-y-z coordinates were imported into the R Geomorph package that was used to calculate Procrustes distances, calculate inter-landmark absolute distances and perform ANOVA statistics to determine significance. For boxplots representing inter-landmark distance measurements, a significant reduction in size of the mutant mandibles is labeled as a percentage of the wild-type mandible. Hotelling tests were performed using Procrustes transformed data, and the hotelling.test function in R. Plotting was performed in R. Numbers of embryos analyzed is indicated in the figure legends.

#### Comparison to other datasets

To determine similarity of the epigenomic landscape at the *SOX9* locus to that of other human cell-types, we downloaded over 50 publicly available H3K27ac ChIP-seq datasets from a number of cell-types (Prescott et al., 2015). We compared these datasets to our *in vitro* hCNCC H3K27ac ChIP-seq data and determined that activity of the PRS-associated enhancer clusters EC1.45, EC1.35 and EC1.25 was restricted to hCNCCs as they were not marked in other cell-types analyzed.

#### Analysis of differentially methylated regions (DMRs)

A Neanderthal-specific significantly hypomethylated region was identified at genomic coordinates chr17:68668482-68674772 (hg19) from previously published datasets (Gokhman et al., 2014, 2020) that overlaps the EC1.45 enhancer cluster. A heatmap was generated representing DNA methylation at CpG dinucleotides spanning the DMR locus for one chimp (rib), seven anatomically modern humans (femur, crania, teeth), one Denisovan (finger) and two Neanderthal (femur and toe) bone samples. DNA methylation levels were determined by whole genome bisulfite sequencing (WGBS) or reconstructed using previously published methods which leverages spontaneous C → T deamination for ancient samples (Gokhman et al., 2014, 2020). For reconstructed DNA methylation maps, the C → T ratio was calculated for each CpG dinucleotide and then translated via a linear transformation (based on modern fully hypomethylated or fully methylated sites) to a methylation percentage. All samples were smoothed using a sliding window of 25 CpG dinucleotides. DNA methylation is marked in the heatmap from green (0% methylation) to red (100% methylation), while white indicates no information.

## Supporting Citations

The following references appear in the Supplemental Information: Day et al. (2009), Limberg et al. (2013), Tawil et al. (1994), Zeevaert et al. (2009).

## Supplemental Information

Document S1. Figures S1–S7 and Tables S1–S4

Table S5. A List of Primers and Probes Used for ChIP-ddPCR, RT-ddPCR, and qRT-PCR Analyses; Primers for Genotyping; and Cloning Guide RNAs and HDR Templates, Capture-C Probes, and Coordinates of PRS Locus Enhancer Loci, Related to STAR Methods

Document S2. Article plus Supplemental Information
