# Supplementary material for: Loss of Extreme Long-Range Enhancers in Human Neural Crest Drives a Craniofacial Disorder
Source: Cell Stem Cell. 2020 Nov 5;27(5):765–783.e14. doi: 10.1016/j.stem.2020.09.001 (PMC7655526; doi:10.1016/j.stem.2020.09.001)
Supplement: Document S1. Figures S1–S7 and Tables S1–S4 [file mmc1.pdf]

Cell Stem Cell, Volume *27*

Supplemental Information

Loss of Extreme Long-Range Enhancers in

Human Neural Crest Drives a Craniofacial Disorder

Hannah K. Long, Marco Osterwalder, Ian C. Welsh, Karissa Hansen, James O.J. Davies, Yiran E. Liu, Mervenaz Koska, Alexander T. Adams, Robert Aho, Neha Arora, Kazuya Ikeda, Ruth M. Williams, Tatjana Sauka-Spengler, Matthew H. Porteus, Tim Mohun, Diane E. Dickel, Tomek Swigut, Jim R. Hughes, Douglas R. Higgs, Axel Visel, Licia Selleri, and Joanna Wysocka

Figure S1, Related to Figure 1

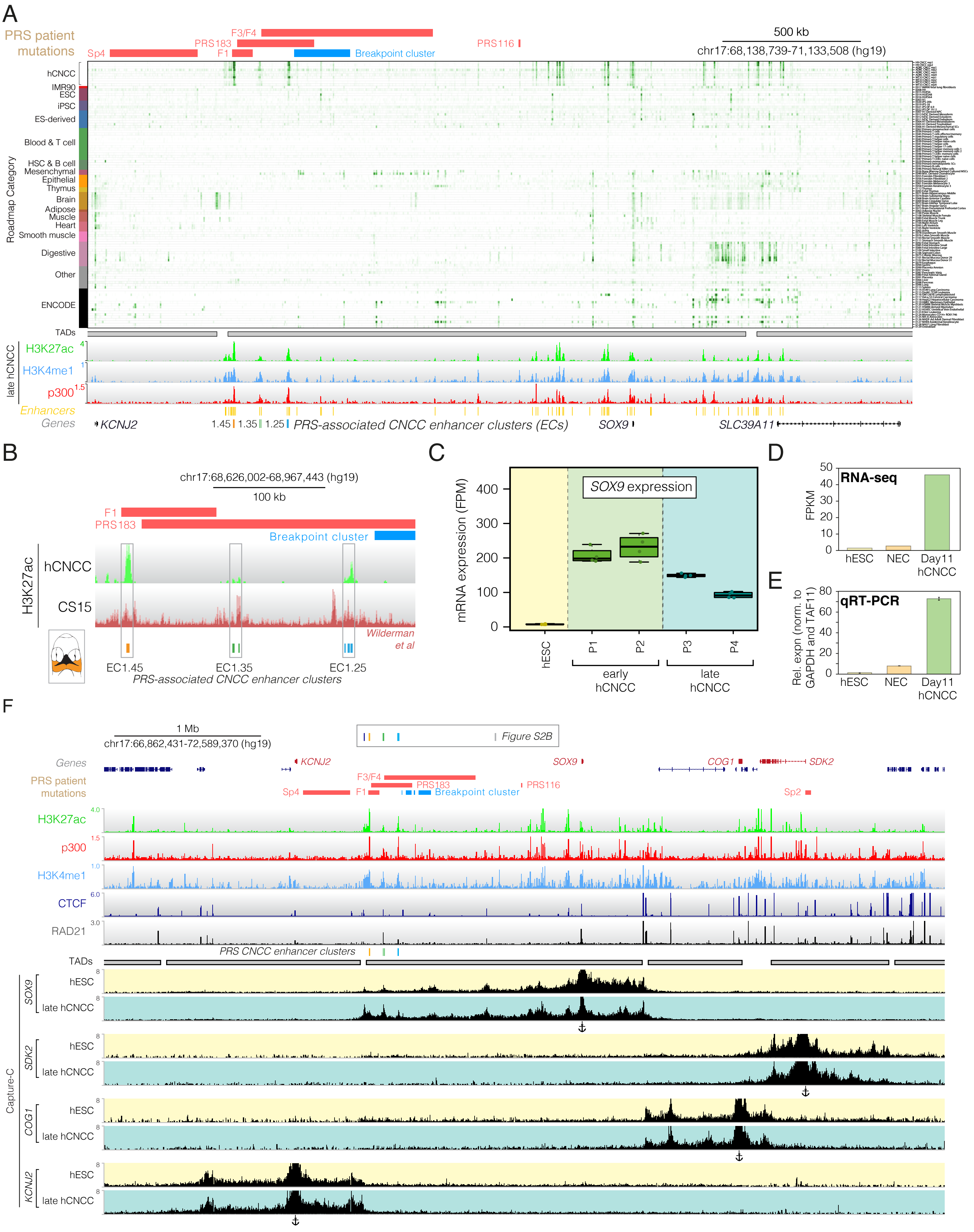

Figure S1. hCNCC-specific enhancer profiles at the *SOX9* PRS locus, induction of *SOX9* expression during hCNCC differentiation and Capture-C interaction profiles for additional craniofacial genes around the PRS locus, **Related to Figure 1**

(A) Publicly available H3K27ac ChIP-seq profiles from the ROADMAP epigenomic project were visualized for many human tissues and compared to profiles from hCNCCs and hESCs. H3K27ac-marked enhancers identified in hCNCCs at the PRS locus appear to be unique to hCNCCs. Enhancers identified in (Prescott et al., 2015) are annotated in yellow. Protein-coding genes are annotated.

(B) H3K27ac ChIP-seq data for dissected human craniofacial tissue at embryonic stage CS15 (Wilderman et al., 2018) compared to *in vitro* derived P4 late hCNCC. H3K27ac coincides with EC1.45, EC1.35 and EC1.25 at CS15 stage of human embryonic craniofacial development.

(C) *SOX9* expression is upregulated around 20-fold during hCNCC differentiation as measured by RNA-seq (fragments per million, FPM).

(D) *SOX9* expression during CNCC differentiation in hESC, neuroectodermal spheres (NEC) spheres and day 11 hCNCCs from published RNA-seq (Rada-Iglesias et al., 2011, 2012).

(E) qRT-PCR for *SOX9* expression in duplicate for the same stages as shown in (C). Data are represented as mean $\pm$ SD.

(F) Capture-C from the viewpoint of genes surrounding the *SOX9* locus associated with craniofacial development. *SDK2* is associated with embryonic skeletal development (Day et al., 2009), while *KCNJ2* and *COG1* loss of function is associated with human craniofacial disorders (Limberg et al., 2013; Tawil et al., 1994; Zeevaert et al., 2009) (genes highlighted in red, see anchors for Capture sites). *KCNJ2*, *COG1* and *SDK2* gene promoters do not interact with the *SOX9* TAD, nor PRS-associated enhancers. ChIP-seq tracks are shown from hCNCC for H3K27ac, p300 and H3K4me1 (to mark enhancers), and CTCF and RAD21. Capture-C is shown for hESC (yellow background, upper) and P4 late hCNCC (teal background, lower) for the *SOX9* promoter, *SDK2* promoter, *COG1* promoter and *KCNJ2* promoter (genomic location chr17:66,846,519-72,577,859, hg19). Capture-C profiles represent the number of normalized unique interactions per restriction fragment. Mutations mapped from PRS patients are highlighted in red (deletions) and blue (translocation breakpoint cluster). Topological domains (TADs) mark self-interacting regions of chromatin (Dixon et al., 2012) and protein-coding genes are annotated. Regions quantified in Figure S2B are highlighted.

Figure S2, Related to Figure 1

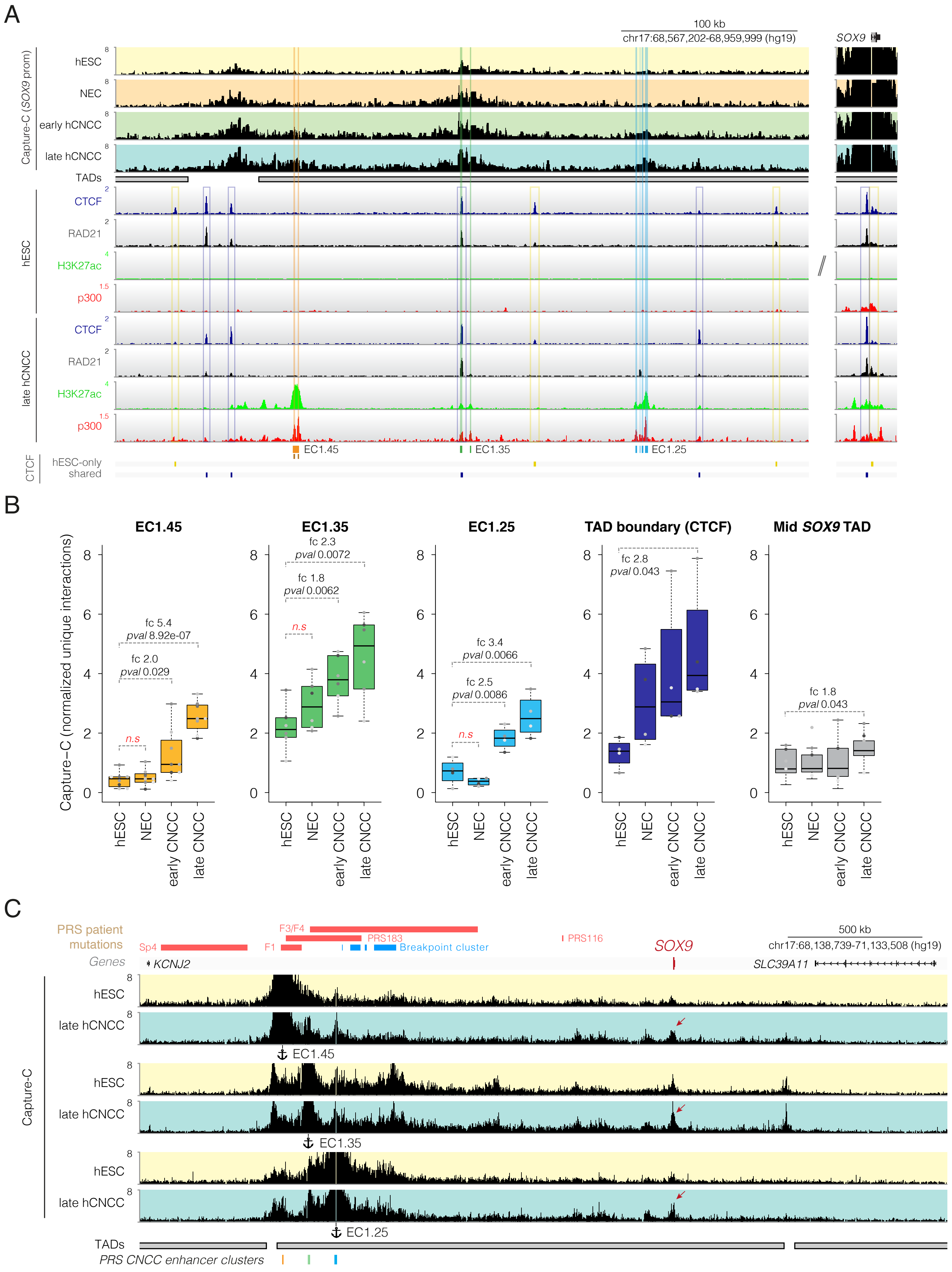

Figure S2. Capture-C interaction profiles for the PRS locus enhancer clusters, **Related to Figure 1**

(A) Capture-C from the viewpoint of the *SOX9* promoter in hESC, neuroectodermal spheres (NEC), early (D11) and late (P4) hCNCCs, focusing on the PRS locus. Long-range interactions can be seen with the three putative enhancer clusters EC1.45, EC1.35 and EC1.25 in late hCNCCs (genomic location chr17:68,567,202-68,959,999, hg19). ChIP-seq data is shown for hESC and hCNCCs including marks of active enhancer (H3K27ac and p300 binding) in addition to CTCF and RAD21. Representative tracks are shown, and CTCF peaks are marked under the tracks as hESC-specific (yellow) or shared between hESC and hCNCC (dark blue). Notably, a CTCF binding site is present in the EC1.35 enhancer cluster and upstream of the *SOX9* promoter in both hESC and late hCNCCs. Enhancer clusters are highlighted in orange (EC1.45), green (EC1.35) and light blue (EC1.25).

(B) Quantification of *SOX9* promoter-anchored Capture-C normalized interaction frequency with restriction fragments at the EC1.45, EC1.35 and EC1.25 loci, along with a CTCF site at the centromeric TAD boundary and a locus in the middle of the *SOX9* TAD. Fold-change of median interaction frequency was determined between hESC and the later stages of the differentiation, and statistical significance determined by t-test. fc, fold-change; pval, p-value from t-test. Sites quantified are highlighted in Figure S1F.

(C) Capture-C from the viewpoint of three distal putative enhancer clusters EC1.45, EC1.35 and EC1.25 for hESC (upper) and P4 hCNCCs (lower). Increased interaction frequency can be seen with the *SOX9* promoter in late hCNCCs compared to hESCs (red arrow, genomic location chr17:68,138,739-71,133,508, hg19). Only protein-coding genes are annotated. Mutations mapped from PRS patients are highlighted in red (deletions) and blue (translocation breakpoint cluster).

Capture-C profiles represent the number of normalized unique interactions per restriction fragment. Topological domains (TADs) mark self-interacting regions of chromatin (Dixon et al., 2012).

Figure S3, Related to Figure 2

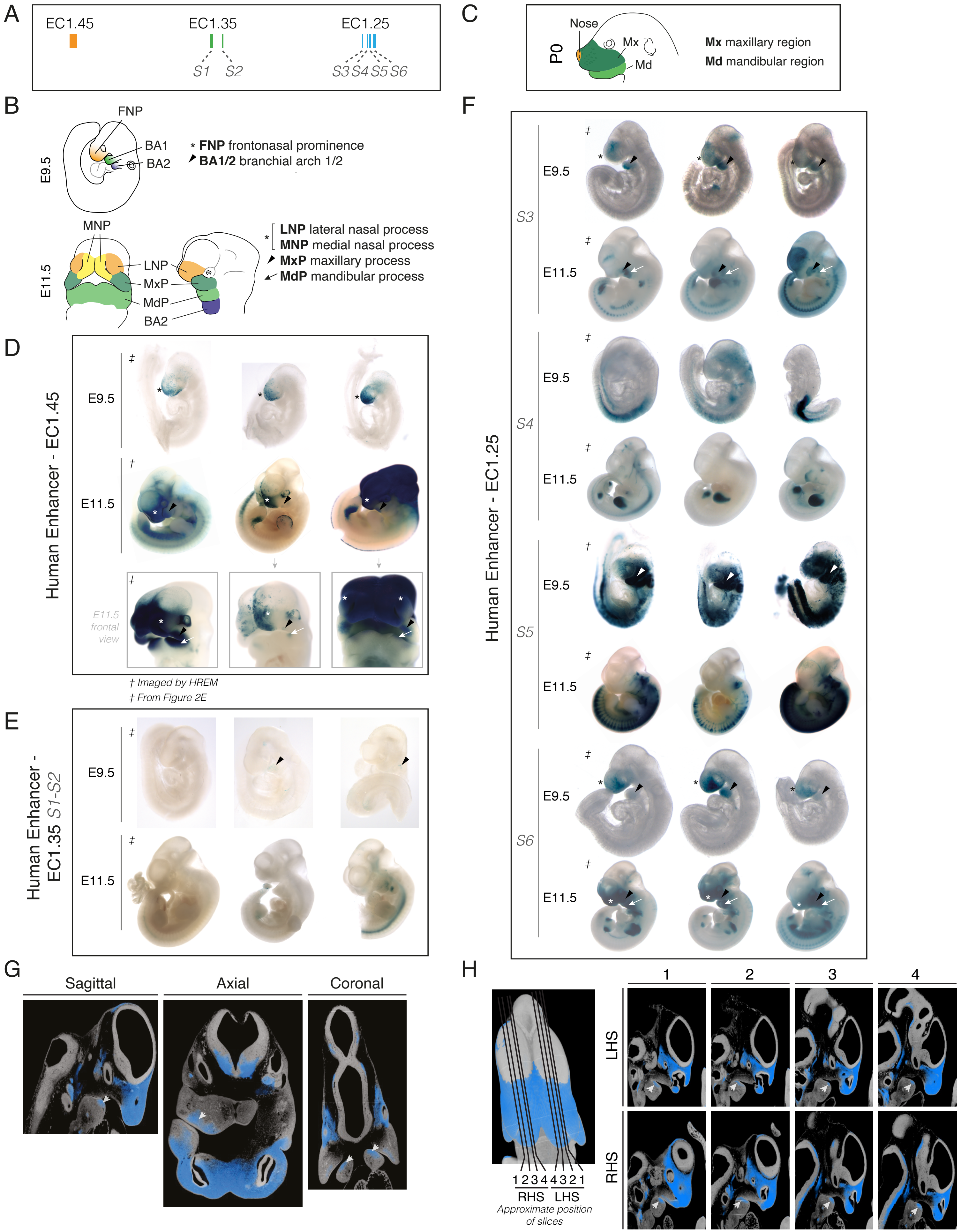

Figure S3. Additional LacZ reporter embryos for enhancer clusters EC1.45, EC1.35 and EC1.25, and additional sections from HREM imaging, **Related to Figure 2**

(A) Schematic of EC1.45, EC1.35 and EC1.25 enhancer clusters and constituent p300 peaks (not to scale).

(B) Schematic outlining craniofacial domains of interest at E9.5 and E11.5. BA1-2, branchial arch 1-2 (arrowhead); FNP, frontonasal prominence (asterisk *); LNP, lateral nasal process (asterisk *); MdP, mandibular process (arrow); MNP, medial nasal process (asterisk *); MxP, maxillary process (arrowhead).

(C) Schematic outlining the components of the face derived from the embryonic domains shown in (B) for P0 pup. Mx, maxillary region; Md, mandibular region.

(D-F) LacZ reporter mouse embryos at E9.5 (upper) and E11.5 (lower) for EC1.45 (D), EC1.35 (S1-S2) (E) and individual p300 peaks for EC1.25 (S3, S4, S5 and S6) (F). † indicates embryo imaged by HREM. ‡ indicates embryos from Figure 2E, repeated here with expression domains annotated.

(G) Sagittal, axial and coronal HREM sections of EC1.45 LacZ activity at E11.5. White arrow highlights activity in the mandibular process.

(H) HREM sections of EC1.45 LacZ activity at E11.5. LacZ signal appears most proximal in the most para-sagittal sections (section 1), in the region that will give rise to the condylar process. White arrow highlights activity in the mandibular process.

Figure S4, Related to Figure 3

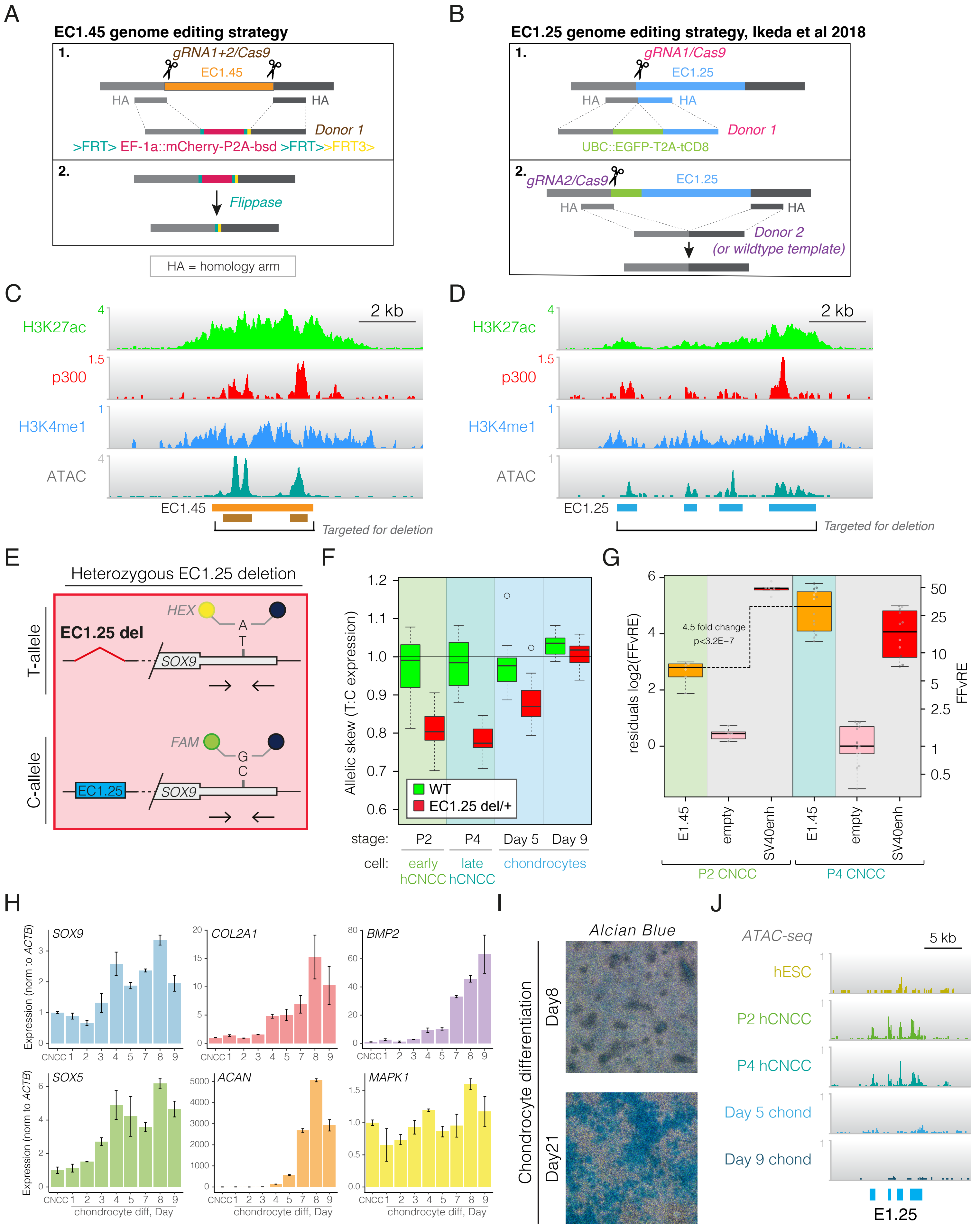

Figure S4. CRISPR/Cas9 genome editing strategy to delete EC1.45 and EC1.25, characterization of chondrocyte differentiation and examination of EC1.25 enhancer activity during hCNCC differentiation, **Related to Figure 3**

(A) A two-step strategy was performed to delete EC1.45. In the first step, a selection cassette (EF-1a promoter driving mCherry-P2A-bsd; bsd = Blasticidin S deaminase), flanked by FRT sites, was inserted in place of EC1.45 by Cas9-mediated homologous recombination in a heterozygous manner. In a second step, Flippase was introduced to induce recombination of the flanking FRT sites, leaving an FRT and FRT3 site at the site of enhancer deletion.

(B) A two-step strategy was performed to generate scar-less deletion of enhancer EC1.25. First, a selection cassette (UBC promoter driving eGFP-T2A-tCD8) was inserted adjacent to the EC1.25 enhancer using Cas9-mediated homologous recombination in a heterozygous manner. In a second step, the cassette was either removed to revert the allele to wildtype, or the cassette was removed along with the enhancer element using using Cas9-mediated homologous recombination.

(C-D) Location of regions targeted for deletion for EC1.45 (C) and EC1.25 (D).

(E) Overview of allele-specific RT-ddPCR for heterozygous EC1.25 deletion indicating primers and locked nucleic acid probes for a T/C SNP in the 3'UTR of the *SOX9* gene in the H9 hESC line. The T-allele is shown above (*in cis* with the EC1.25 deletion), and the C-allele below. A HEX LNA probe will detect the T-allele, and a FAM LNA probe will detect the C-allele.

(F) ddPCR for EC1.25 heterozygous deletion and wildtype lines, using primers and probes as outlined in Figure 3B and (E). Relative expression between the two alleles is plotted (T:C ratio). Reduction in *SOX9* expression for the allele with EC1.25 deletion is observed at all stages of hCNCC differentiation, while allelic expression differences are not detected for Day 9 chondrocytes.

(G) Luciferase reporter assays for EC1.45 at P2 and P4 of the CNCC differentiation indicating a 4.5 fold-change in activity between the two stages.

(H) Gene expression detected by qRT-PCR for hCNCCs and the first 9 days of chondrocyte differentiation. Data are shown for a representative experiment, error bars calculated from technical replicates (n=2). Data are represented as mean $\pm$ SD.

(I) Alcian Blue staining of hCNCC-derived chondrocytes at two stages of chondrocyte differentiation (Day 8 and Day 21).

(J) ATAC-seq for hCNCC differentiation demonstrating hCNCC-specific accessibility for the EC1.25 enhancer.

Figure S5, Related to Figure 4

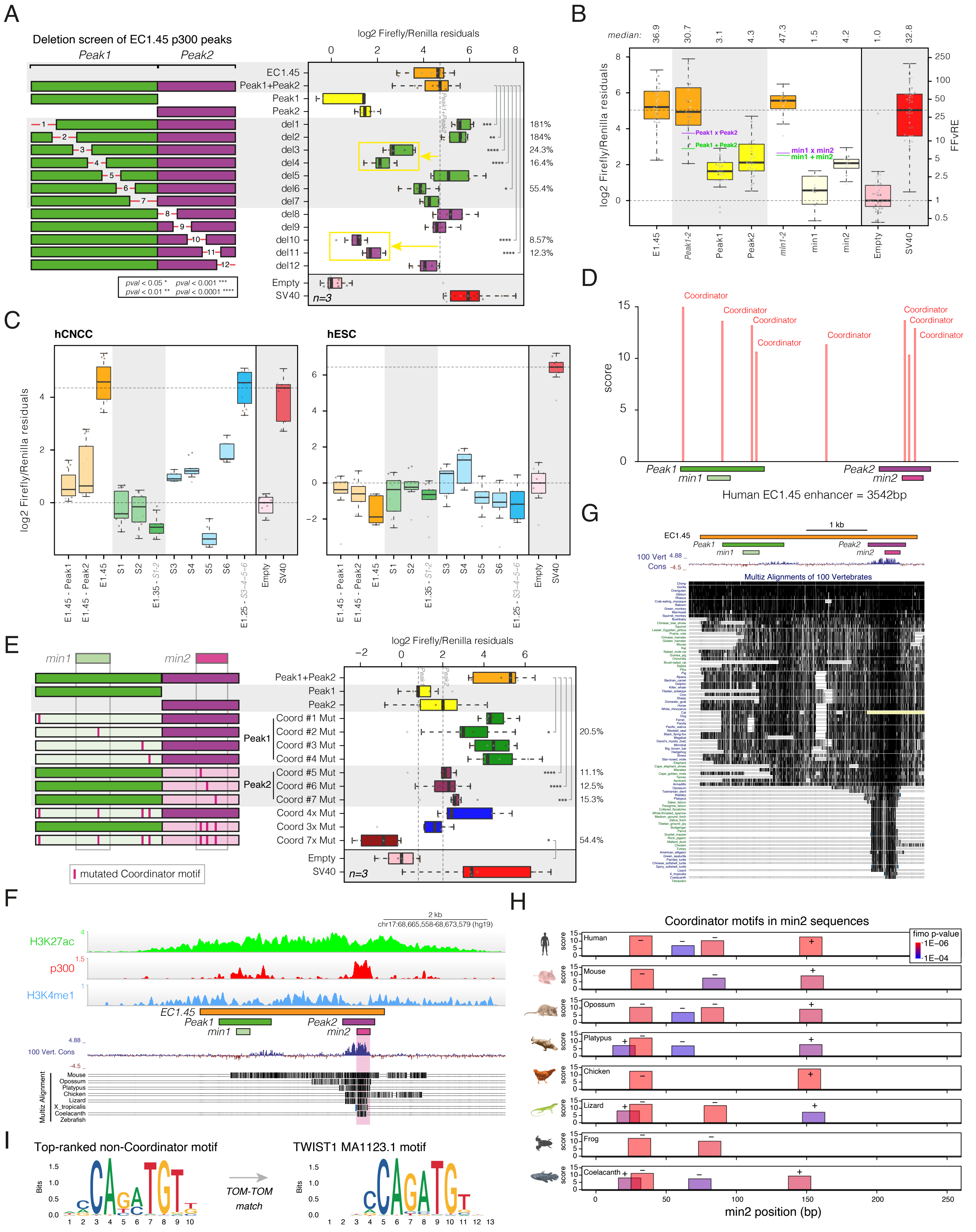

Figure S5. Identification of sequences central to EC1.45 enhancer cluster activity that act in a synergistic manner and are conserved down to lobe-finned fish, coelacanth, **Related to Figure 4**

(A) Luciferase assay for the entire EC1.45 enhancer cluster and subsets thereof. p300 Peak1 and Peak2 were assayed together and separately, and a tiling deletion screen of overlapping ~250bp regions was performed to identify critical regions for enhancer activity. Schematic of tested sequences are depicted on the left. Deletions 3 and 4 within Peak 1, and deletions 10 and 11 within Peak 2 dramatically reduce activity (see yellow boxes), while deletions 1 and 2 within Peak 1 increase activity. Percentage change in median value compared to Peak1+Peak2 is indicated for constructs with a significant change.

(B) Luciferase assay illustrating the synergy between EC1.45 constituent enhancer elements. A horizontal line indicates the expected signal for a sum (green, +) and multiplication (purple, x) of Peak1 and Peak2 or min1 and min2. In both cases the actual combined enhancer activity is greater than expected for multiplicative interaction. Data from 11 independent experiments are plotted.

(C) *In vitro* luciferase reporter assays for p300 peaks from PRS locus putative enhancer clusters tested individually and in combination in human cranial neural crest cells (hCNCC, left) and human embryonic stem cells (hESC, right). Constructs tested from EC1.45 (Peak1, Peak2 and EC1.45), EC1.35 (S1, S2 and S1-2) and EC1.25 (S3,S4,S5, S6 and S3-S4-S5-S6).

(D) Annotation of Coordinator motifs in EC1.45 enhancer detected by fimo. p300 Peak1 and Peak2, along with min1 and min2 sequences are indicated.

(E) Luciferase assay for EC1.45 p300 Peak1 and Peak2 with single or multiple Coordinator mutations. Individual mutations in Coordinator #2, #5, #6 or #7 reduce enhancer activity, and mutation of 4x Coordinator motifs in Peak 1 or 3x motifs in Peak 2 reduce activity to a level similar to Peak2 or Peak 1 respectively. Mutation of all 7 Coordinator sequences lead to a reduction of activity 1.84 fold lower that the empty vector control (t-test, pval = 0.027). A schematic on the left illustrates the constructs tested, with a pink vertical line indicating a Coordinator mutation. Percentage change in median value compared to Peak1+Peak2 is indicated for constructs with a significant change.

(F) Multiz alignment at the human EC1.45 enhancer sequence for selected species from mouse to coelacanth reveals deep conservation of min2 sequence, highlighted in pink.

(G) Multiz alignment for 100 vertebrates highlights conservation of the EC1.45 enhancer and min2 sequence.

(H) Detection of Coordinator sequences for human, mouse, opossum, platypus, chicken, lizard, frog and coelacanth min2 sequences by fimo, plotting score with p-value indicated by color and strand annotated as +/-.

(I) The top non-Coordinator sequence motif enriched at TWIST1 ChIP-seq peaks genome-wide in hCNCCs. TOM-TOM analysis identifies this motif as closely resembling the consensus TWIST1 motif (MA1123.1, JASPAR) (Gupta et al., 2007; Khan et al., 2018).

For luciferase assays, empty vector and SV40 enhancer are negative and positive controls, respectively. Biological replicates are plotted as residuals of linear regression, normalized to the empty vector control. Significance determined by t-test: * pval < 0.05; ** pval < 0.01; *** pval < 0.001; **** pval < 0.0001.

Figure S6, Related to Figures 5 and 7

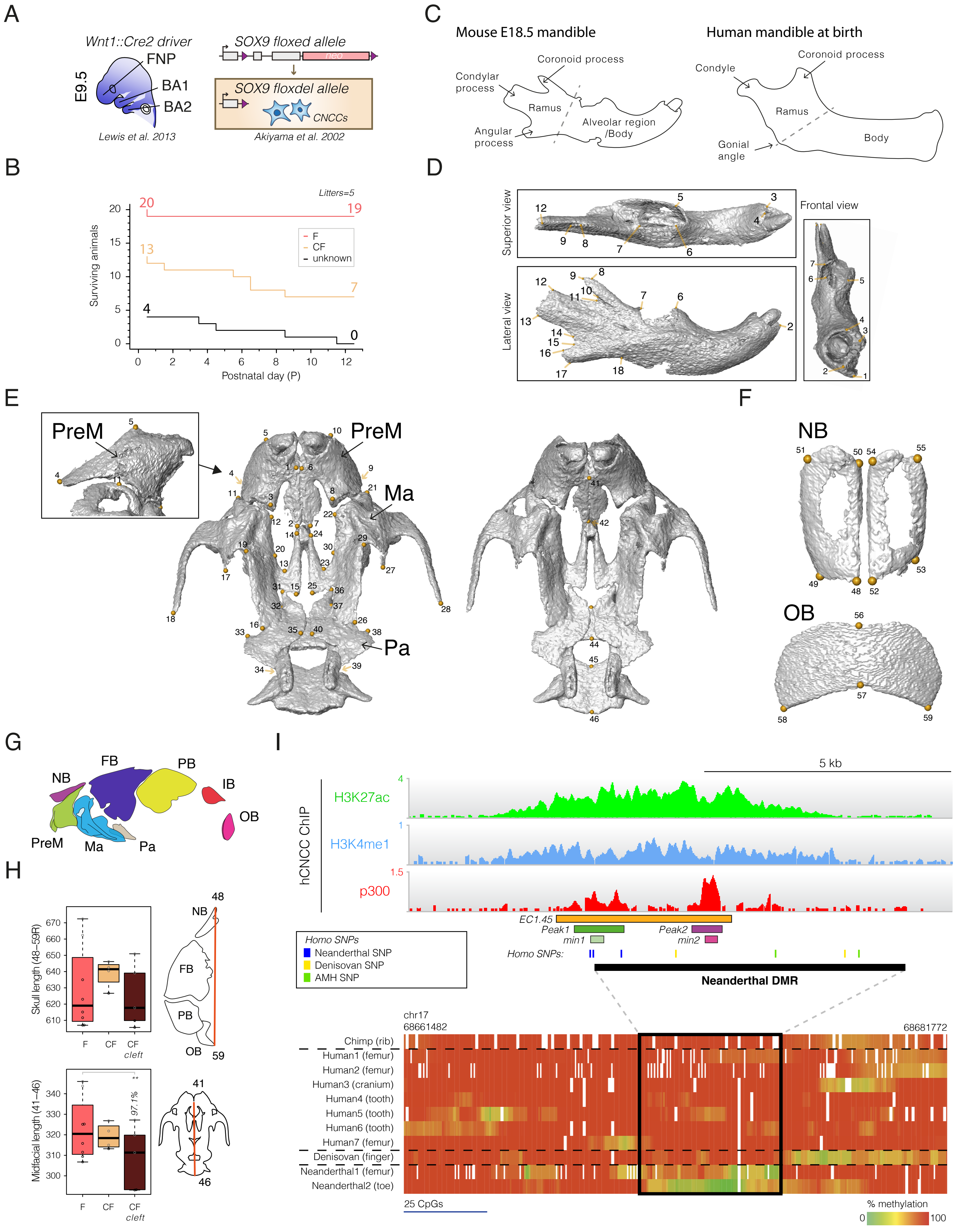

Figure S6. Survival curve for *Sox9* conditional mutant mice, schematics of mandibular and skull morphometric landmarking and a heatmap of a Neanderthal-specific hypomethylated region overlapping the EC1.45 locus, **Related to Figures 5 and 7**

(A) Schematic of *Wnt1::Cre2* driver, and *Sox9* flox allele illustrating location of loxP sites and the resultant floxed allele in the cranial neural crest and derivatives. BA1-2, branchial arch 1-2; FNP, frontonasal prominence. Purple triangles, loxP sites; neo, neomycin resistance.

(B) Survival curve of pups in the postnatal period P0-P12, illustrating that a large number of mutant *Wnt1::Cre2;Sox9F/+* (CF) pups die compared to wildtype *Sox9F/+* (F) siblings during this period.

(C) Schematic of a mouse hemimandible at E18.5, highlighting coronoid, condylar and angular processes (left), compared to a human mandible at birth (right) highlighting the orthologous structures coronoid process, condyle and gonial angle.

(D) Right mouse hemimandible at E18.5, illustrating the location of 18 mandibular landmarks. Mirrored landmarks were also placed on the left hemi mandible.

(E) Bones of the mouse midface at E18.5, illustrating the location of 46 landmarks

(F) Nasal bones (upper) and occipital bone (lower) at E18.5, illustrating the location of 8 and 4 landmarks respectively.

(G) Schematic of skull bones from a lateral viewpoint, including those used for landmarking.

(H) Boxplot of distance measurements for wildtype (*Sox9F/+*, F) and conditional *Sox9* mutant (*Wnt1::Cre2;Sox9F/+*) skulls with (CF cleft) and without (CF) clefting, showing skull length and midfacial length.

(I) ChIP-seq traces for enhancer marks at the EC1.45 enhancer cluster in hCNCC. EC1.45 and minimal active regions min1 and min2 are indicated along with an overlapping Neanderthal-specific differentially methylated region (DMR) (upper). Homo SNPs are indicated, including three Neanderthal SNPs in blue. A heatmap depicts DNA methylation levels along the EC1.45 locus, color-coded from green (unmethylated) to red (methylated), where white indicates no information (lower). This region is specifically hypomethylated in Neanderthal samples (44-58%) and methylated in AMH (82%-99%), chimpanzee (83%) and Denisovan (88%) samples.

FB, frontal bone; IB, interparietal bone; Ma, maxilla; NB, nasal bone; PB, parietal bone; OB, occipital bone; Pa, palatine bone; PreM, premaxilla.

Figure S7, Related to Figure 6

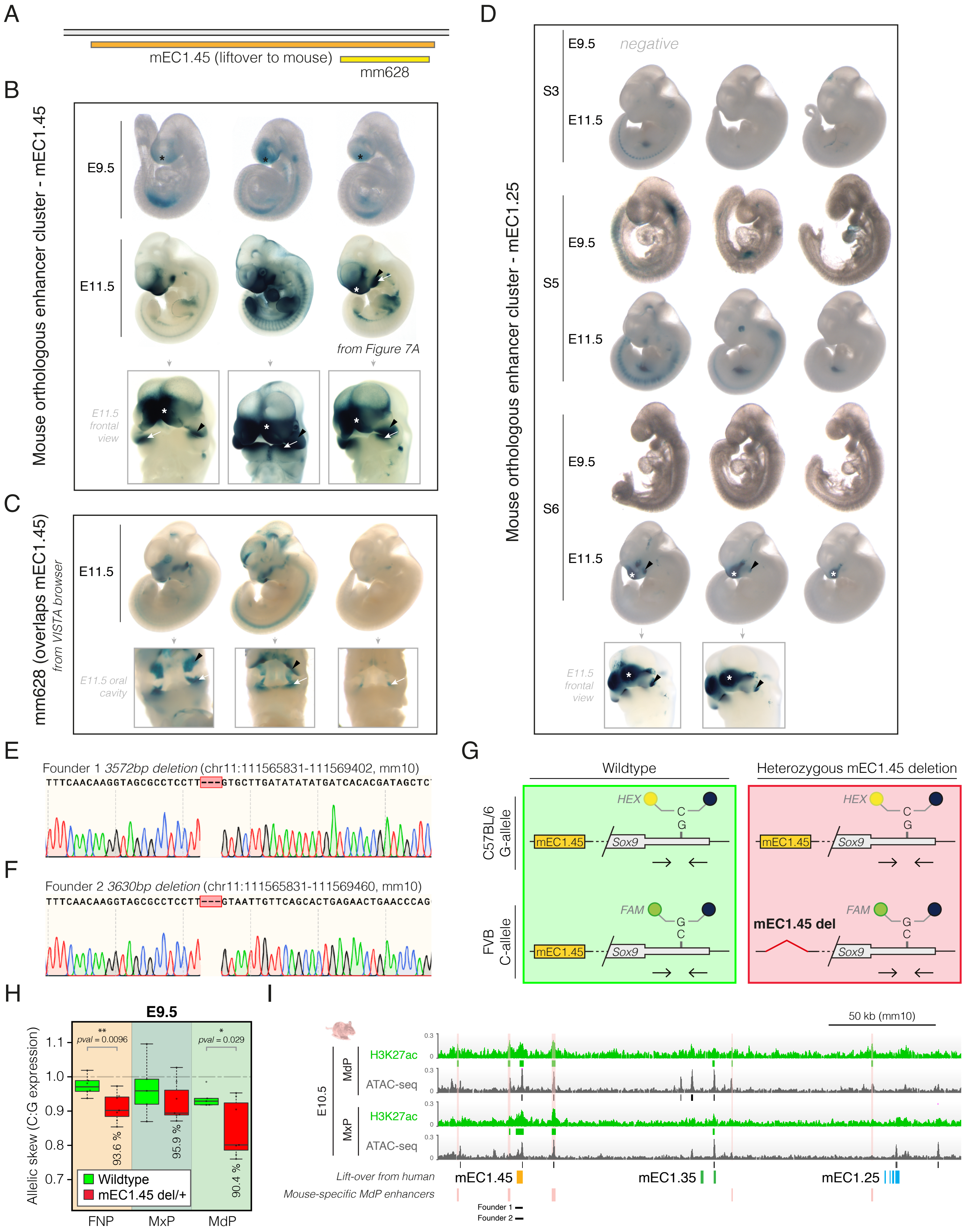

Figure S7. LacZ reporter embryos for orthologous mouse enhancer clusters mEC1.45 and mEC1.25 and validation of mEC1.45 deletion, **Related to Figure 6**
(A) Schematic of mouse orthologous mEC1.45 locus showing the location of the VISTA browser mm628 element (Visel et al., 2007).
(B) LacZ reporter mouse embryos at E9.5 (upper) and E11.5 (lower) for mouse orthologous mEC1.45. Insets of E11.5 embryos show frontal-lateral views for visualization of craniofacial domains.
(C) LacZ reporter mouse embryos at E11.5 for mouse sequence mm628 initially reported in VISTA enhancer browser (Visel et al., 2007). Insets show oral cavity.
(D) LacZ reporter mouse embryos at E9.5 (upper) and E11.5 (lower) for mouse orthologous mEC1.25 p300 peaks S3, S5 and S6. Insets of E11.5 embryos show frontal-lateral views for visualization of craniofacial domains.
(E-F) Sanger sequencing traces of genomic deletions from Founder 1 (E) and Founder 2 (F) mouse lines.
(G) Overview of allele-specific RT-ddPCR indicating primers and locked nucleic acid probes for a G/C SNP in the 3'UTR of the *Sox9* gene for the C57BL/6 (G-allele) and FVB (C-allele) mouse strains. The G-allele is shown above, and the C-allele below, with wildtype alleles depicted on the left, and the heterozygous mEC1.45 deletion on the right. A HEX LNA probe will detect the G-allele, and a FAM LNA probe will detect the C-allele. In a cross between wildtype C57BL/6 and *mEC1.45del/+* FVB animals, 50% of resulting embryos will be heterozygous null for the mEC1.45 enhancer cluster on the FVB allele (*Sox9* C-allele).
(H) Digital droplet RT-PCR for *Sox9* from mEC1.45 heterozygous deletion and wildtype dissected E9.5 craniofacial tissues (21-27 somites). Relative *Sox9* expression for the two alleles is plotted as C:G ratio. Significant reduction in *Sox9* expression is observed for the FVB C-allele (with mEC1.45 deleted) for the FNP and MdP compared to the C57BL/6 G-allele. Significance determined by t-test, * pval < 0.05; ** pval < 0.01.
(I) Epigenomic profiles of putative enhancers from CNCCs sorted from mouse craniofacial tissues at E10.5 (Minoux et al., 2017). Lift-over of human enhancer clusters highlight the orthologous mouse mEC1.45, mEC1.35 and mEC1.25 regions. Peaks were called from H3K27ac ChIP-seq data, and putative enhancers (beyond those overlapping the lift-over regions) from mouse mandibular process CNCCs are highlighted in pink. The region deleted in mEC1.45del Founders 1 and 2 is indicated.
FNP, frontonasal prominence; M/LNP, medial and lateral nasal processes; MxP, maxillary process; MdP, mandibular process.
For E9.5 LacZ reporter embryos, an asterisk indicates FNP. For E11.5, an asterisk indicated M/LNP; arrowhead MxP; arrow MdP.

Supplemental Tables

Table S1. Number of LacZ positive embryos at E9.5 and E11.5, **Related to Figures 2 and 6**

	**Stage**	
**Construct**	**E9.5**	**E11.5**
Human EC1.45	7	4
Human EC1.35 (S1-2)	5	3
Human EC1.25 S3 x3	4	4
Human EC1.25 S4 x3	3	5
Human EC1.25 S5 x3	8	4
Human EC1.25 S6 x3	5	4
Mouse mEC1.45	3	9
Mouse mEC1.25 S3 x3	0 *	5
Mouse mEC1.25 S5 x3	3	3
Mouse mEC1.25 S6 x3	4	5

The number of LacZ positive embryos from the LacZ reporter assay at E9.5 and E11.5. * No LacZ positive embryos were detected from 3 PCR positive embryos. Constructs that were triplexed are indicated with “x3”.

Table S2. Reproducible activity domains for LacZ reporter embryos, **Related to Figures 2 and 6**

	Stage and Tissue						
	E9.5			E11.5			
**Construct**	**FNP**	**BA1**	**Other**	**LNP/ MNP**	**Mx**	**Md**	**Other**
Human							
EC1.45	7/7			4/4	4/4	4/4	4/4 HL;4/4 FL; 4/4 POM
EC1.35 (S1-2)	NONE /5			NONE /3			
EC1.25 S3 x3	4/4	4/4			4/4	4/4	4/4 DRG; 3/4 mesenchyme near tail *
EC1.25 S4 x3			3/3 trunk mesoderm				5/5 FL; 5/5 HL
EC1.25 S5 x3		8/8	6/8 BA2; 6/8 somites; 4/8 IM				4/4 DRG; 4/4 TG; 4/4 gut tube
EC1.25 S6 x3	5/5	4/5		3/5	4/5	4/5	3/5 BA2; 4/5 FL; 4/5 HL
Mouse							
mEC1.45	5/5		4/5 FL	8/9	8/9	8/9	8/9 FL; 7/9 HL
mEC1.25 S3 x3	NONE /3			NONE /5			
mEC1.25 S5 x3	NONE /3						3/3 abdomen
mEC1.25 S6 x3			4/4 few migratory cells in BA1/2	5/5	3/5		

LacZ positive embryos are scored for domains of activity at E9.5 and E11.5. Expression domains are considered reproducible if they are detected in 3 or more embryos (highlighted in blue). Constructs that were triplexed are indicated with “x3”. BA1, branchial arch 1; BA2, branchial arch 2; DRG, dorsal root ganglia; FL, forelimb; FNP, frontonasal prominence; HL, hindlimb; IM, intermediate mesenchyme; MdP, mandibular process; LNP, lateral nasal process; MNP, medial nasal process; MxP, maxillary process; POM, periocular mesenchyme; TG, trigeminal ganglia; NONE, no reproducible expression domains. * LacZ activity likely in anus and genital tubercle.

Table S3. Phasing information for H9 hESCs at *SOX9* locus, **Related to Figure 3**

	Near EC1.45	Near EC1.45	Near EC1.45	Near EC1.25	*SOX9* UTR
SNP	rs2009802	rs2009779	rs3952733	rs2367117	rs74999341
Haplotype 1	T	C	A	A	T
Haplotype 2	C	T	G	G	C

Phased SNPs in H9 hESCs from 10X Genomics linked-read sequencing adjacent to the EC1.45 and EC1.25 enhancer clusters (used for genotyping enhancer deletion lines), and in the 3'UTR of the *SOX9* gene. For heterozygous H9 deletion cell lines the enhancer cluster is deleted on Haplotype 2 for EC1.45del/+ lines, and Haplotype 1 for EC1.25del/+ lines.

Table S4. Measurements of PRS patient mandibles, **Related to Figure 5**

Study	Patients, mean age in months *mo*, or years *yr* (SD)	Mean ramus height, mm (SD) *PRS vs control*	Mean mandibular body length, mm (SD) *PRS vs control*
Volk, 2020	24 **isolated PRS** 0.6mo; 24 control 0.7mo	16.7 (1.9) vs 17.3 (1.6); *P = .346* ↓	35.3 (3.3) vs 39.3 (3.2); *P < .001* ↓
Zellner, 2017	15 PRS (including **syndromic**) 1.1mo; 15 control 1.3mo	19.7 vs 22.7 ↓	35.2 vs 37.2 ↓
Chen, 2015*	5 **PRS (no syndrome defined)** 0.7mo (0.5); 4 control 4.5mo (4.3)	17.3 (2.6) vs 23.0 (2.7) ↓	27.6 (3.0) vs 40.6 (4.0) ↓
Bienstock, 2016	5 PRS (**not specified if syndromic**) 2.2mo; 5 control 12.2mo	21.0 vs 30.0 ↓	35.6 vs 45.2 ↓
Susarla, 2018	11 PRS (**including Stickler syndrome**) 8.9mo (6.4); 9 control 8.4mo (6.4)	27.0 (4.1) vs 32.0 (4.5) (no p-val) ↓	-
Chung, 2012*	5 PRS (**not specified if syndromic**) 38.4mo (51.5); 6 control 60.0mo (24.0)	40.9 (6.3) vs 44.4 (3.5) ↓	48.8 (7.3) vs 59.4 (4.0); *P < .05* ↓
Suri, 2010*	34 **isolated PRS** 11.7yr (0.7); 34 control 11.9yr (0.8)	50.4 (4.7) vs 53.9 (3.5) *P < 0.001*↓ (Co-Go)	65.2 (4.3) vs 75.0 (3.54) *P < 0.001*↓ (Go-Gn)

SD, standard deviation; studies ordered by mean age at time of imaging; *, poor age-match
